# CRISPR/Cas9: a sustainable technology to enhance climate resilience in major Staple Crops

**DOI:** 10.3389/fgeed.2025.1533197

**Published:** 2025-03-18

**Authors:** Navjot Kaur, Muslim Qadir, Dali V. Francis, Anshu Alok, Siddharth Tiwari, Zienab F. R. Ahmed

**Affiliations:** ^1^ Department of Integrative Agriculture, College of Agriculture and Veterinary Medicine, United Arab Emirates University, Al-Ain, United Arab Emirates; ^2^ College of Agriculture, South China Agricultural University (SCAU), Guangzhou, Guangdong, China; ^3^ Department of Plant Pathology, University of Minnesota, Saint Paul, MN, United States; ^4^ Plant Tissue Culture and Genetic Engineering Lab, BRIC-National Agri-Food and Biomanufacturing Institute (BRIC-NABI) (Formerly National Agri-Food Biotechnology Institute), Department of Biotechnology, Ministry of Science and Technology (Government of India), Mohali, Punjab, India

**Keywords:** climate-smart crops, food security, genome editing, maize, rice, wheat

## Abstract

Climate change is a global concern for agriculture, food security, and human health. It affects several crops and causes drastic losses in yield, leading to severe disturbances in the global economy, environment, and community. The consequences on important staple crops, such as rice, maize, and wheat, will worsen and create food insecurity across the globe. Although various methods of trait improvements in crops are available and are being used, clustered regularly interspaced short palindromic repeats and CRISPR-associated protein 9 (CRISPR/Cas9) mediated genome manipulation have opened a new avenue for functional genomics and crop improvement. This review will discuss the progression in crop improvement from conventional breeding methods to advanced genome editing techniques and how the CRISPR/Cas9 technology can be applied to enhance the tolerance of the main cereal crops (wheat, rice, and maize) against any harsh climates. CRISPR/Cas endonucleases and their derived genetic engineering tools possess high accuracy, versatile, more specific, and easy to design, leading to climate-smart or resilient crops to combat food insecurity and survive harsh environments. The CRISPR/Cas9-mediated genome editing approach has been applied to various crops to make them climate resilient. This review, supported by a bibliometric analysis of recent literature, highlights the potential target genes/traits and addresses the significance of gene editing technologies in tackling the vulnerable effects of climate change on major staple crops staple such as wheat, rice, and maize.

## 1 Introduction

The world is facing drastic climate change, with high global temperatures and elevated carbon dioxide (CO_2_) levels. This has resulted in extreme events, adversely affecting all dimensions of the world, including agriculture, biodiversity, and human community. The root cause of climate change is greenhouse gas emissions, mainly from anthropogenic activities, ascending the global surface temperature by 1.5°C since 1850 (Karavolias et al., 2021; Nunez et al., 2019). Recent reports confirmed that the year 2023 was the hottest in Earth’s history, which had several consequences such as drought, compound flooding, heavy precipitation, global sea rise, and upper sea acidification in certain regions of the planet (Li Z. et al., 2024). Rising temperatures are a warning sign for current agricultural production, although they have already started devastating effects worldwide. Climate change will mostly affect crop yield at lower latitudes more than at higher latitudes (Shukla et al., 2019). The situation of agriculture at lower latitudes might worsen with a temperature rise, whereas higher latitudes might benefit from higher temperatures, which would increase crop yield (Iizumi et al., 2018). More specifically, areas closer to the equator are more prone to desertification and eventual agricultural loss, which has already started in the Asian and African continents (Zougmoré et al., 2018). These regions are already facing high populations and unsustainable land management issues; therefore, their agricultural productivity and biodiversity are under great threat due to climate change (Viana et al., 2022). Increasing temperatures rise to over 35°C in California caused the browning of berries, reducing the yield by almost 50% (Kizildeniz et al., 2018).

The current scenario indicates that crop production in tropical areas will be most affected by high temperatures and droughts (Esquivel-Muelbert et al., 2019). It is estimated that food crop production in Africa will be reduced by 2.9% by 2050 owing to climate change. Global productivity losses disrupt other facets of the ecosystem.

The entire ecosystem relies on food supplies, occupying the highest agricultural land share. Studies have revealed that approximately 90% of food calories and 80% of the proteins and fats originate from agricultural land. Hence, agricultural land contributes to food security and various FAO sustainability goals (Avtar et al., 2020; FAO, 2017). However, most arable land has been degraded because of non-sustainable agricultural practices, including spraying chemical fertilizers, excess groundwater use, intensive farming, and deforestation. Such practices have increased greenhouse gas emissions, which are a major cause of temperature increases (Funk, 2021; Shahzad et al., 2021). Therefore, some areas of the globe might experience drought while others might be flooded owing to rising sea levels. Currently, areas suitable for crop production will soon become unsuitable (Iizumi et al., 2018). Therefore, identifying suitable areas for crop production is crucial for addressing the impacts of climate change. Several studies have focused on identifying suitable areas for agriculture in different countries (Musakwa, 2018). However, this alone is insufficient to overcome the effects of climate change.

A meta-analysis of about a hundred studies explained the impacts of climate change on biodiversity and found that a moderate rise in temperature can cause significant harm to biodiversity (Nunez et al., 2019). Owing to climate change pressure, food production needs to be enhanced, which requires more land, exacerbating biodiversity loss. For instance, the production of soybeans, palm oil, beef, and wood from 2000 to 2011 in seven countries was responsible for 40% of the deforestation of tropical forests and carbon losses (Henders et al., 2015; Ortiz et al., 2021).

**FIGURE 1:**
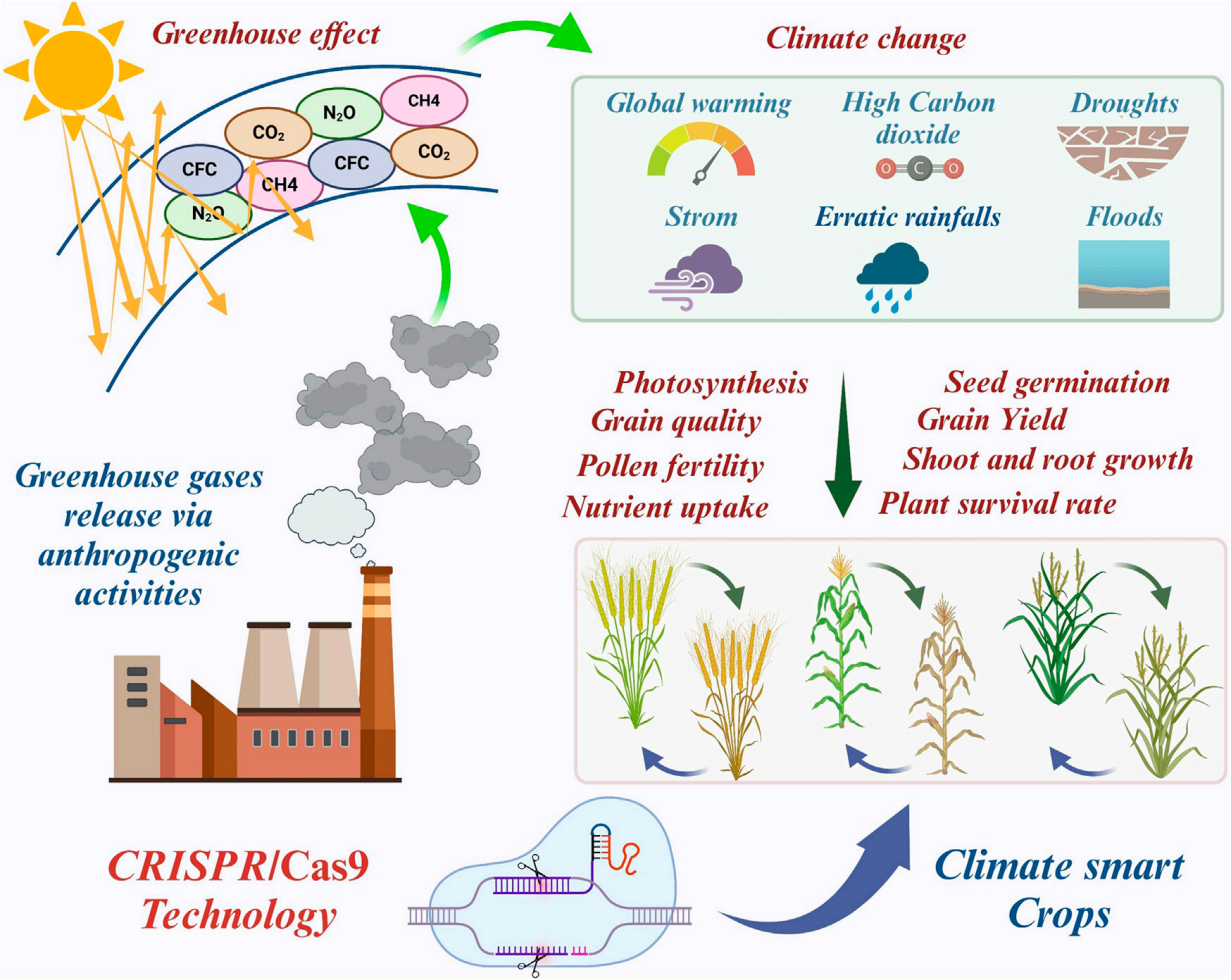
Gene editing, a sustainable technology to reduce the Vulnerability of major Staple Crops to Climate Change (Graphical abstract).

Despite this, plants adapt extraordinary mechanisms to survive in the harsh climate. Such mechanisms involve root and leaf modification, stomatal regulation, osmotic adjustment, ion transport and sequestration, morphological behavior, and genetic adaptations. However, these processes require years to develop a climate-resilient plant (Krishna et al., 2023). Therefore, dealing with these issues in a short step is feasible using a genome-editing technique called clustered regularly interspaced short palindromic repeats and CRISPR-associated protein 9 (CRISPR/Cas9). Climate-smart crops in terms of increasing abiotic and biotic stress tolerance and high-yielding biofortified crops can be generated using this approach (Figure 1). Here, we summarize the progression in crop improvement from conventional breeding methods to advanced genome editing techniques and how the CRISPR/Cas9 technology can be applied to safeguard the main cereal crops (wheat, rice, and maize) from harsh climates.

## 2 Evolution of crop improvement from conventional breeding, and genetic engineering to genome editing

**FIGURE 2:**
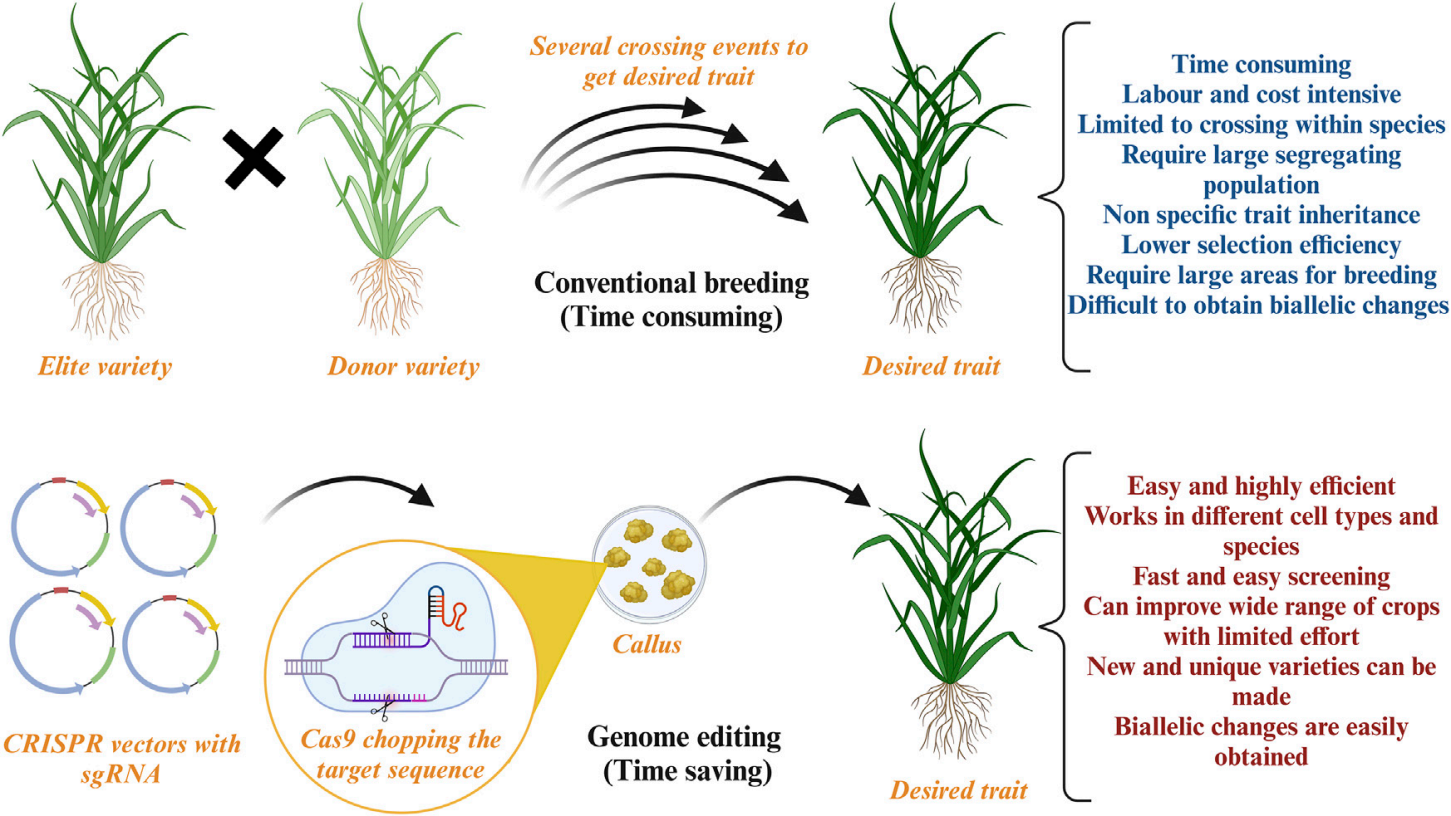
Conventional breeding versus Genome editing: This figure illustrates the advantages of genome editing over conventional breeding techniques. Created with BioRender.

Although climate change is progressing at an exceptional rate, it is not easy to envisage the loss it can cause to agriculture. Nevertheless, the scientific community has made tremendous efforts with conventional crop improvement techniques to combat the effects of climate change. Such approaches include breeding that produces superior varieties using donor and recipient plants with desired characteristics (Sharma et al., 2023; Van et al., 2022). This process was revolutionized in 1940–1950 when semi-dwarf wheat varieties were developed. Although hybrid varieties are superior and popular innovations, reaching the final product is time-consuming, costly, and requires intensive labor. Breeding is associated with the plant’s phenotypic trait, which is highly influenced by environmental factors, and requires several backcrosses to obtain the desired trait. Furthermore, conventional breeding methods result in selecting an inferior parent crop limiting the germplasm gene pool and causing genetic erosion (Krishna et al., 2023). Breeding can lead to the development of undesired traits because the transfer of genetic information cannot be controlled (Figure 2). Additionally, large arable land requirements with huge investments are another drawback of conventional breeding. The scientific community is making tremendous efforts using breeding to combat the effects of climate change. However, these efforts are insufficient, and more advanced strategies are required to improve agricultural techniques.

Later, the emergence of recombinant DNA technology, where the genetic material of plants could be modified by inserting a foreign gene of interest to produce superior transgenic crops, also called genetically modified (GM) crops, changed the concept of producing superior varieties. Therefore, genetic manipulation can be performed in a controlled manner. Currently, GM crops, including BT cotton, corn, and soybean, are utilized worldwide, especially in the United States of America (USA) (Wechsler, 2018). Because this approach directly deals with transferring genetic information from one species to another, some myths addressing biodiversity and health concerns remain controversial. In addition, a long procedure that requires clinical trials and money expenditure is required to launch a GM product in the market (Van et al., 2022). Likewise, similar trials are required for genetically engineered plants produced via RNA interference (RNA*i*) technologies because of its several disadvantages, including, off-targeting effects that may lead to plant toxicity, development of insect resistance, incomplete or variable levels of silencing, and highly programmatic designing process (Sharma et al., 2023).

Gene-editing technologies have attracted the attention of the scientific community owing to their simplicity for designing, efficient editing, and accuracy. Genome editing technology uses molecular scissors to create double-stranded breaks in DNA, and the remaining part is undertaken by the host DNA repair machinery, which can either add or remove nucleotides randomly, leading to the formation of mutants. This modification can be achieved through site-specific insertion-deletion (indels), substitution, or epiallelic changes within the targetted DNA in a cell or organism. Genome editing is based on the principle of DNA repair, in which strand breaks are introduced using molecular scissors, such as endonucleases (Carroll, 2014), mega-nucleases (Gong and Golic, 2003), zinc finger nucleases (ZFNs) (Urnov et al., 2005), transcription activator-like effector nucleases (TALENs) (Sun and Zhao, 2013), and CRISPR/Cas9 (Jinek et al., 2012).

Among the above-mentioned methods CRISPR/Cas endonuclease based is the most popular, which has gained momentum in the last 10 years owing to its high efficiency, ease of use, and accuracy. CRISPR/Cas systems are diverse and adopted from bacteria and archaea. Currently, there are many tools such as CRISPRi, CRISPRa, base editor, gene knock-in, targeted protein tagging, and Viral mediated editing, that arose from the basic CRISPR/Cas9 system (Anzalone et al., 2020; Kampmann, 2018). Variants of Cas endonuclease, i.e., dCas9-foki, Cas9 nickase, HypaCas9, Sniper-Cas9, eSpCas9 (1.1), SpCas9-HF1, xCas9, evoCas9, SuperFi-Cas9, miCas9, evoCjCas9, SpRYc, KG, and SpdNG-QT.12j (Goldberg et al., 2021; Jeon et al., 2018; Karvelis et al., 2021; Kulcsár et al., 2022; Ma et al., 2020; Schmidheini et al., 2024; Schmidt et al., 2021; Schuler et al., 2022; Sun A. et al., 2023; Thakur et al., 2024; Wang et al., 2021a; Zhao et al., 2023). Moreover, Cas9 functional analogs such as, Cas12a-b, Cas12d-f, Cas12h-j, Cas121, Cas12n, Cas12 λ, Cas13 (C2c2), and Cas14 have been developed that extend the editing capability to the RNA and protein levels to enhance the performance of this technique (Hillary and Ceasar, 2022; Schindele et al., 2018; Yan et al., 2019). These all tools have been successfully applied to various crops for different purposes to alter the metabolic pathway (Ahmar and Gruszka, 2023; Kaur et al., 2020; Li D. et al., 2024; Ly et al., 2024; Toinga-Villafuerte et al., 2022; Wang J. D. et al., 2024; Xie Y. et al., 2024).

**FIGURE 3:**
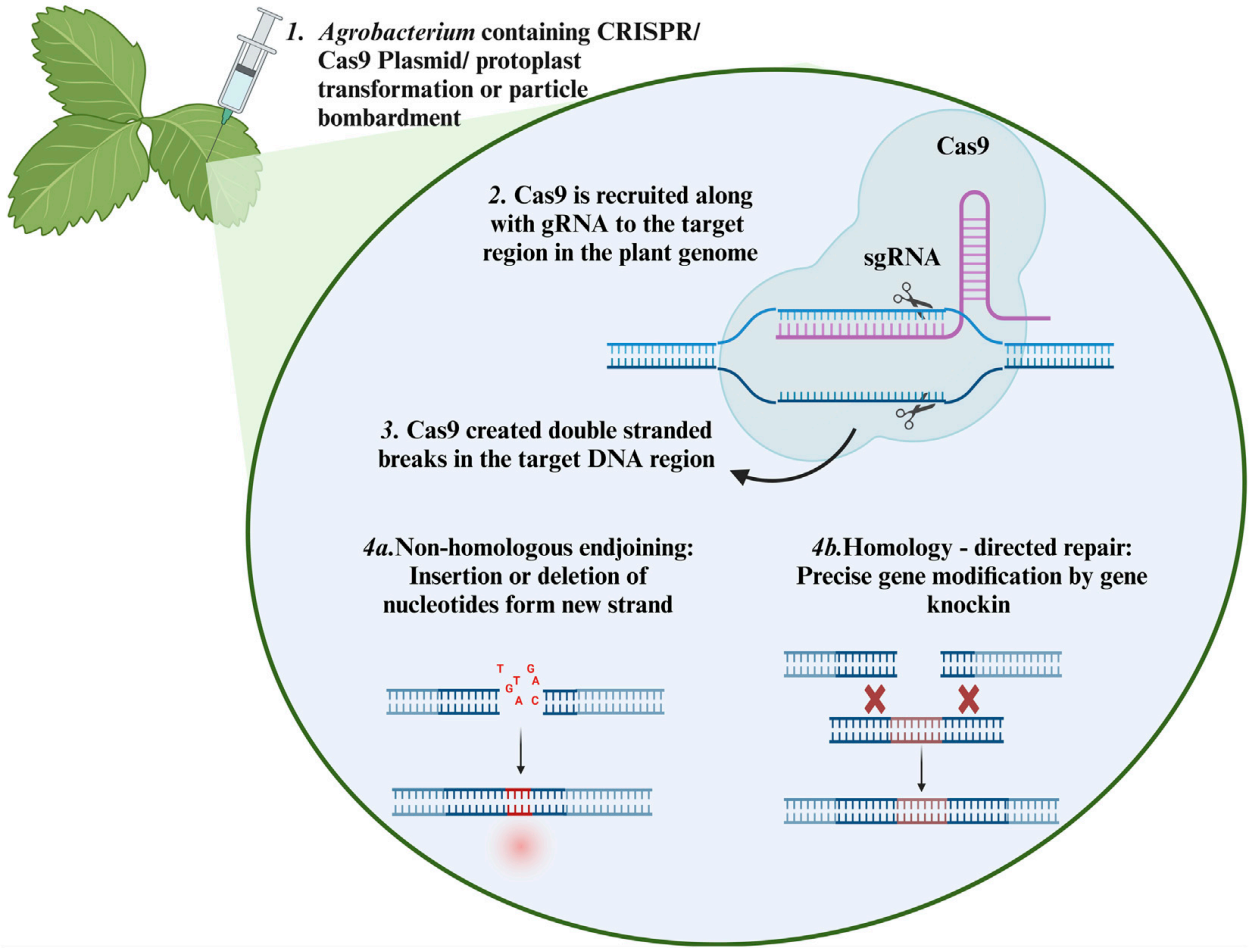
Mechanism of CRISPER/Cas9 in the plant: (1) The CRISPR/Cas9 system can be introduced into plant cells by *Agrobacterium* mediated transformation, protoplast transformation or particle bombardment. (2) The sgRNA guides the Cas9 to the target region of the genome. (3) Cas9 recognize the PAM region and creates double stranded cut. (4a) InDels are generated in the target site through the NHEJ repair system (4b) Precise corrections can be made in the DNA or directed sequences can be inserted through HDR repair system Created with BioRender.

The detail mechanism of CRISPR/Cas9 for generating knockout events in plants is described in Figure 3. It has been applied to gene editing and transcriptional modulation of plants to improve various agronomical characteristics, such as drought tolerance, salinity tolerance, heat stress tolerance, disease resistance, nutritional enhancement, and yield improvement. Because plant phenology is affected by climate change events, this technology can also be used to control plant development-related factors for instance, flowering, male sterility, and photoperiod) (Cai et al., 2018; Liu et al., 2019; Shen et al., 2017). CRISPR/Cas9 derivatives, including prime and base editors, also provide opportunities to modify the plant genome precisely. Substantial research is currently underway to improve these techniques.

Therefore, considering the vast potential of gene editing technologies, they may be the best solution for mitigating the effects of climate change (Singh et al., 2024a). This review describes the impact of climate change on food security and how it can affect the main cereal crops (wheat, rice, and maize) that are highly prone to climate change, and the research initiatives for their improvement via the CRISPR/Cas9 approach are also discussed in this review. The applications of this technology are extensive; however, certain limitations such as government regulations must be considered.

## 3 Climate changes impact on major staple crops and improvement strategies using CRISPR/Cas9

### 3.1 Wheat: improvement of traits via CRISPR/Cas9 approach

Wheat is an important cereal crop that serves two purposes: feed for the global community and support for the nation’s economy. Average wheat production worldwide is over 700 million tons, with China, India, the United States, the Russian Federation, and France being the top five wheat producers. The main exporters of wheat are the United States, Canada, France, Australia, and Russia, whereas the main importers are Egypt, Italy, Brazil, Japan, and Algeria (http://faostat.fao.org/). The overall production and trade of wheat reflects its significant role and demand in the global population. Approximately 95% of the total wheat produced worldwide is hexaploid bread wheat (*Triticum aestivum* sp. *Aestivum*, AABBDD, 2n = 6x = 42), whereas the remaining 5% is tetraploid durum wheat (*Triticum turgidum* sp. *Durum*, AABB, 2n = 4x = 28), also known as pasta wheat. The dough-forming ability of wheat flour increases the product range of wheat into bread, pasta, noodles, and biscuits. The key components responsible for dough formation are the grain storage proteins called gluten in wheat flour, whose interactions with water upon kneading form a proteinaceous structure (Shewry, 2019). Starch is another important component of wheat that promotes dough formation and causes gelatinization. In total, mature wheat grains contain approximately 13% water, 71% carbohydrates, 11% proteins, 2% lipids, 2% minerals, and 0.1% vitamins and phytochemicals, contributing significantly to human health (Wieser et al., 2020). Wheat has undoubtedly contributed significantly to global food security; however, wheat production is at great risk owing to climate change. As a C3 crop, wheat may benefit from high CO_2_ concentrations in the environment by improving water-use efficiency, photosynthesis, and transpiration. However, the grain quality can be negatively affected by higher levels of CO_2_. At higher temperatures, wheat productivity may decline because of a shorter crop season and an increase in transpiration (Bouras et al., 2019). A report published in Nature Climate Change estimated the impact of rising temperatures on global wheat yield using three independent models. The results indicated that the per degree rise in temperature can decline wheat productivity by 4.1%–6.4% (Liu et al., 2016). Various studies have shown that increasing temperature can have a drastic effect on wheat grain quality by shortening the grain-filling period, which will affect the gluten composition of wheat. High temperatures can change the ratio of gliadin to glutenin, weakening the dough-making properties of wheat flour. Moreover, extended heat waves and high temperatures can reduce the nitrogen level and, ultimately, the protein content of the grain (Wang and Liu, 2021b). In conclusion, climate change is going to impact wheat; therefore, the development of climate-tolerant wheat, which is difficult through conventional breeding techniques due to its hexaploidy genome, is an utmost priority. CRISPR/Cas9 technology was successfully used to obtain stable inherited mutations in wheat. *Agrobacterium*-mediated transformation has been used to deliver CRISPR constructs into immature wheat embryos (Howells et al., 2018; Zhang Z. et al., 2019). CRISPR/Cas9 genome editing-derived transgene-free wheat plants have also been produced using biolistic and protoplast transfection methods (Liang et al., 2017; Zhang et al., 2016). These studies have successfully implemented CRISPR/Cas9 genome editing technology in wheat, providing a platform for improving wheat concerning climate change-mediated problems. Grain weight, grain size, and grain yield per plant are the main agronomic attributes highly influenced by abiotic and biotic factors. Researchers have identified several genes that function as negative regulators of grain’s weight, size, and overall yield. For example, *Receptor-like protein kinase 1* (*RPK1*), the *Brittle rachis gene* (*BTR1-A*), *GASR7*, *TaSPL13*, *GW2*, and *TaARE1* (Table 1). A study reported the knockout of *GASR7* and *GW2* genes via the ribonucleoprotein (RNP)-derived CRISPR/Cas9 approach in bread wheat and pasta wheat resulted in transgene-free mutants with higher grain weight (Zhang Y. et al., 2021). Similarly, the *TaSPL13* gene, responsible for controlling grain size and number, was edited using CRISPR/Cas9 (Gupta et al., 2023). The results revealed that mutations in this gene lead to an increase in the size and number of grains in allohexaploid wheat, demonstrating the importance of *TaSPL13* in the evolution of yield-related attributes in wheat. Similarly, *Ta-eIF4E* alone has been targeted in wheat, resulting in viral resistance and improved plant height and grain length (Kan et al., 2023). Another important agronomic factor in wheat is early heading. Recently, the role of the *thioredoxin gene* (*TaTRXH9*) is characterized and validated in wheat using CRISPR/Cas9-mediated gene editing, and a loss-of-function mutation in this gene found in early-heading wheat (Fan et al., 2023). These studies reveal the success of CRISPR/Cas9 genome editing in improving wheat yield, which can significantly mitigate the impact of climate change on yield reduction.

**TABLE 1:** CRISPR/Cas9-mediated gene editing in Wheat.

Sr No.	Targeted genes	Role in plant	Knockout effects on plant	References
1	*TtMTL*	Haploid induction is triggered mainly by *MATRILINEAL* (*MTL*)	Haploid production	Chang et al. (2024)
2	*TaRPK1*	*Receptor-like protein kinase 1* (*RPK1*) has been reported to regulate root architecture system (RAS), abiotic stress, and yield	Enhanced yield	Rahim et al. (2024)
3	*TaPPD-1*	It influences spike development by affecting late and earlier flowering	Increase spike architecture and grain	Errum et al. (2023)
4	*TaTRXH9*	Associated with heading time regulation	Early heading	Fan et al. (2023)
5	*TaSPL13*	It represents important targets for improving grain yield and other major agronomic traits in rice	Increased grain size and number	Gupta et al. (2023)
6	*TaSPO11-1*	Involves in programmed meiosis-specific DNA double-strand breaks during recombination	Restore crossover sites, synapsis, and fertility leading to increased seed set	Hyde et al. (2023)
7	*TaARF12*	Involves in auxin pathway	Reduce plant height with larger spikes shows higher grain yield	Kong et al. (2023)
8	*TaARF15-A1*	*TaARF15-A1* as a negative regulator of senescence in wheat	Accelerated leaf senescence and grain ripening	Li et al. (2023a)
9	*TaPPO1, TaPPO2*	*PPOs* are dual-activity metalloenzymes that catalyze the production of quinones, discolor of flour, dough, and end-use products	Develop grain and vegetative tissues	Wold-McGimsey et al. (2023)
10	*TaMYB10*	Activates flavonoid biosynthesis genes to specify red grain color and influences	Seed coat permeability is reduced in variants, and is associated with PHS tolerance of grains	Zhu et al. (2023)
11	*TaFT-D1*	It significantly associated with the total spikelet number and heading date	Rise in total spikelet number	Chen et al. (2022d)
12	*TaIPK1*	Involves in the final step of the phytate biosynthesis pathway	Improved the iron and zinc content and lowered the phytic acid accumulation in wheat grains	Ibrahim et al. (2022)
13	*TaSBEIIa*	Starch-branching enzyme	High amylose content varieties	Li et al. (2021b)
14	*TaARE1*	A negative regulator of nitrogen assimilation	Increase grain yield and quality	Zhang et al. (2021b)
15	*TaPINb-47*	Genes control grain texture and hardness	Harder wheat grains	Zhang et al. (2021c)
16	*TaPP O -7*	It catalyzes phenol oxidation into dark-colored products	Improved grain color	
17	*TaPSY-13*	Involves in carotenoid biosynthesis	Low downstream metabolites	
18	*TaWAXY-2*	Involves in amylose synthesis in wheat endosperm	Glutinous wheat produced with lower amylose content	
19	*TaGW7*	Encodes TRM protein affects grain shape and weight in allohexaploid wheat	Shorter and wider grains	Wang et al. (2019)
20	*TaCKX2-D1*	Grain-regulatory genes	Increased grain number per spikelet	Zhang et al. (2019b)
21	*TaGLW7*	Grain-regulatory genes	Increase wheat grain size and grain weight	
22	*TaGW2*	Grain-regulatory genes	Increased grain weight as well as protein content	
23	*TaGW8*	Grain-regulatory genes	Increase wheat grain size and grain weight	
24	*α- or γ-gliadins*	Possesses immunogenic epitopes for celiac disease	Developed hypoimmunogenic-gluten” wheat lines	Jouanin et al. (2019)
25	*TaAT1*	Associated with regulating the levels of reactive oxygen species (ROS) and stress-related signaling pathways	Increased salt-alkaline tolerance, heightened plant growth, and decreased ROS accumulation	Sun et al. (2023b)
26	*TaSAL1*	Negative regulator of drought tolerance	Improves drought tolerance	Mohr et al. (2022)
27	*TaMBF1c*	Confers thermotolerance by regulating specific mRNA translation	Upregulated in response to heat stress	Tian et al. (2022)
28	*TaHAG1*	Contribute to salt tolerance by modulating ROS production and signal specificity	Enhanced salt tolerance	Zheng et al. (2021a)
29	*TaMKP1*	Defense against devastating fungal pathogens and determined its subcellular localization	Enhanced resistance to rust and powdery mildew in wheat	Liu et al. (2024c)
30	*TaGRF4*	GRF4 protein and its interacting factor (GIF1) to develop a reproducible genetic transformation and regeneration protocol	Resistance against leaf rust and powdery mildew	Biswal et al. (2023)
31	*TaCIPK14*	CBL-interacting protein kinases are involved in defense responses during plant-pathogen interactions	Increased wheat resistance to *Puccinia striiformis* fungus	He et al. (2023)
32	*TaeIF4E*	Encodes a cap-binding protein binds to methylated guanine triggering assembly of protein translation initiation complex	Viral resistance, improvement in plant height and grain length	Kan et al. (2023)
33	*TaMLO-A1*	*Mildew resistance locus O* (*MLO*), susceptibility gene	Resistant against Powdery mildew fungus	Li et al. (2022b)
34	*TaHRC*	*Histidine-rich calcium-binding protein* gene (*TaHRC*) as the gene for Fhb1, a major quantitative locus for FHB resistance	Suppresses the calcium-mediated immune response and triggers wheat *Fusarium head blight* susceptibility	Chen et al. (2022a)
35	*TaBAK1-2*	Encodes *BRI1-associated receptor kinase 1*—an important regulator of plant immunity and development	Increased immunity toward virus	Hahn et al. (2021)
36	*Ta-eIF4E* and *Ta-eIF(iso)4E*	Translation-initiation factors serve as susceptibility (*S*) factors required for plant viruses from the Potyviridae family to complete their life cycle	Viral resistance	

The unstable weather can increase gluten levels in wheat, which makes it less suitable for consumption (Mkhabela et al., 2022). However, Chinese researchers have developed a Gluten gene Enrichment and Sequencing (GlutEnSeq) system, screened thousands of *prolamin* genes from different wheat varieties, and low gluten wheat was produced by modifying *γ*- and *a-gliadin* genes via CRISPR/Cas9 (Jouanin et al., 2019). A study developed improved winter and spring wheat varieties with high amylose content via CRISPR/Cas9-mediated genome editing of starch-branching enzyme (SBE) II (*TaSBEIIa*), and multiple transgene-free mutant lines were obtained (Li J. et al., 2021). Correspondingly, the CRISPR/Cas9 approach has been utilized to modify four genes, i.e., *puroindoline b* (*PINb*), *granule-bound starch synthase gene* (*GBSS* or *WAXY*), *polyphenol oxidase* gene (*PPO*), and *phytoene synthase* gene (*PSY*), in wheat via the *Agrobacterium*-mediated transformation method of gene delivery (Zhang Y. et al., 2021; Zhang Z. et al., 2019). The *PINb* gene controls grain hardness, the *WAXY* gene is responsible for amylose synthesis, *PSY* is the main gene controlling the carotenoid biosynthetic pathway, and *PPO* controls color via the oxidation of phenolic compounds.

Micronutrient deficiency in wheat is likely to occur because of increasing temperatures and drought, which can lead to malnutrition and other health disorders. The CRISPR/Cas9 genome editing approach is a straightforward method that can be used to enhance the nutritional composition of wheat and address this problem; for instance, Ibrahim et al. edited the *Inositol Pentakisphosphate 2-Kinase 1* (*TaIPK1*) gene, which expresses an enzyme involved in the final step of the phytate biosynthesis pathway (Ibrahim et al., 2022). Phytic acid is considered an anti-nutrient because it reduces the bioavailability of iron and zinc in humans. The phytic in wheat tissues binds to these micronutrients as phytate and decreases their bioavailability. CRISPR/Cas9 genome engineering of *TaIPK1* in wheat improved iron (1.5–2.1-fold) and zinc content (1.6–1.9-fold) and lowered phytic acid accumulation in wheat grains. Several agronomic improvements via CRISPR/Cas9-mediated genome editing in wheat are presented in Table 1.

Transcriptome profiling of wheat grains revealed several heat stress-associated gene, including heat shock transcription factor gene (*TaHSFA6e*), *ascorbate peroxidase*, *β-amylase*, *γ-gliadin-2*, and *LMW-glutenin*, were upregulated during the high-temperature stress (Rangan et al., 2020; Wen et al., 2023). Such studies can help select the potential target genes for CRISPR/Cas-mediated genome editing, which might lead abiotic stress resistant crops. Moreover, studies on different crops can be used to develop wheat that is tolerant to increasing temperatures caused by climate change. For example, CRISPR/Cas9-mediated knockout of the *mitogen-activated protein kinase* gene (*SlMAPK 3*) enhances heat tolerance in tomato plants, and the edited *pyrabactin resistance 1* (*PYR1*)/*PYR1*-*like* (*PYL*) (*pyl1/4/6*) makes rice tolerant to hot weather (Miao et al., 2018; Yu et al., 2019). Since wheat is hexaploid and has a complex genome, there are fewer reports available on producing abiotic stress-tolerant wheat plants than rice. *TaSAL1*, *TaMBF1c*, and *TaHAG1* genes have been edited to produce abiotic stress-tolerant wheat plants (Mohr et al., 2022; Tian et al., 2022; Zheng M. et al., 2021). The *histone acetyltransferase* (*TaHAG1*) gene contributes to salt tolerance by affecting free radical production in hexaploid wheat. *MBF1c* confers thermotolerance by regulating the translation of specific mRNA translation, whereas *SAL1* negatively regulates drought tolerance (Table 1). These studies suggest that genome editing of wheat could be a promising approach for conferring climate change.

### 3.2 Rice: improvement of traits via CRISPR/Cas9 approach

Rice (*Oryza sativa L*.) is a staple crop, with a global consumption rate of approximately 21% and 76% in Asian countries. Worldwide, 776.5 million tons of rice were produced in 2022, with approximately 90.5% of the average rice production taking place in Asia, in which China and India are the biggest producers (). This high demand for rice is due to its taste and versatility in a variety of international cuisines (Castanho et al., 2023). In addition, rice is enriched in nutrients, mainly complex carbohydrates, and moderate levels of vitamin B, phosphorous, iron, calcium, and protein. Most nutrients (minerals, vitamins, proteins, and antioxidants) are present in the rice brain, which has a brown outer layer. Rice contains all essential amino acids except lysine and is a great source of a balanced diet, as it does not contain cholesterol, fat, or sodium (Sasaki and Burr, 2000). However, the current climate change scenarios have adversely affected rice crops from farms to consumers in various ways (Zhao et al., 2016). Increasing temperature, carbon dioxide, drought, salinity, rainfall, pests, and diseases are the main stressors that can directly affect various rice attributes, such as grain size, quality, yield, nutritional constitution, and appearance. Rice cultivation can be successful if the optimum temperature and rainfall are provided; however, an increase of 1 °C can adversely affect rice yield. A recent study showed a decline in the nutritional content of rice due to rising atmospheric CO_2_ concentrations. Micronutrients, proteins, and several vitamins are diminished in rice, leading to malnutrition in infants and children of rice-dependent countries. A decline of approximately 17%–30% in vitamins, 5% in zinc, 8% in iron, and 10% in protein was observed in rice grown under high CO_2_ conditions (Smith and Myers, 2018). In contrast, Guo et al. found a 15% increase in the mineral content of rice grown under high CO_2_ and temperature conditions (Guo et al., 2022). Though high CO_2_ is normally considered a growth-stimulating agent along with high temperature, it acts antagonistically. Jing et al. reported reduced rice yield due to elevated temperatures under high CO_2_ conditions created using temperature-free air CO_2_ enrichment (T-FACE) systems (Jing L. et al., 2024). Another T-FACE experiment in China reported that increasing CO_2_ substandardized the sensory quality of rice by increasing chalkiness (Wang et al., 2024d). The chalkiness of rice is a major obstacle to rice marketing. Chalky rice forms owing to abnormal starch development inside the grain and appears as a scattered coarse material, thus making the surface turbid. This abnormal starch accumulation occurs due to the high temperature, mainly at the time of grain filling, which reduces the ability of α-amylase enzyme to degrade the starch and, hence, leads to malformed starch synthesis (Shimoyanagi et al., 2021). In addition to grain quality, other issues in rice that can occur due to climate change include decreased shoot biomass and carbohydrate content in the stem, decreased starch content, and reduced photosynthetic ability, transpiration, and leaf area (Cui et al., 2024; Huanhe et al., 2024; Yamori et al., 2025). Hence, concerning the future outcomes, researchers have been trying different strategies, one of which is the robust and versatile genetic editing technique called as CRISPR/Cas9 system for generating climate-smart rice crops. CRISPR/Cas9-mediated gene editing has been optimized in rice using *Agrobacterium*-mediated transformation, particle bombardment, and RNPs (Miao et al., 2013; Shan et al., 2013). Moreover, a multiplexing approach was successfully established for rice. Recently, a group of researchers accomplished ultra-multiplexing targeting 49 genes in rice using both *Agrobacterium*-mediated and biolistic approaches (Wu et al., 2024).

CRISPR/Cas9 mediated-knockout of two main quantitative trait loci associated with grain length and thousand-grain weight, i.e., *GS3* and *GL3.1* was reported in rice *GS3* encodes a protein that restrains the cell division of the spikelet hull, leading to shorter grains, whereas *GL3.1* encodes an enzyme that controls grain size by dephosphorylating the cell cycle-related protein Cyclin-T1,3 and inhibits cell proliferation in the hull. Mutations in this gene increase grain size in rice (Yuyu et al., 2020; Zhang Y. M. et al., 2021). Huang et al. used the CRISPR/Cas9 technique to create an Indica maintainer line, Mei1B, containing an edited *GS3* allele to improve grain yield and quality (Huang et al., 2022). Another study mutated the *OsSPL16* or *GW8* gene via CRISPR/Cas9 technology to improve the cylindrical shape and grain yield of Basmati rice (Usman et al., 2021). Rice aroma is an important quality parameter that is affected by climate change. Nevertheless, improving and introducing aromas into rice is feasible to improve and introduce aromas into rice using genetic engineering techniques. Mutations in *Betaine Aldehyde Dehydrogenase 2* (*OsBADH2*) via CRISPR/Cas9 added aroma to elite non-aromatic rice variety (Hui et al., 2022; Tang et al., 2021). Several reports are available on the use of CRISPR-mediated gene editing for improving various agronomic traits in rice (Table 2). Recently, loss-of-function mutants of *OsCKX* were found to affect various attributes including plant height, grain size, grain number, panicle size, seed shape, and starch accumulation. This gene encodes a cytokinin-degrading enzyme that inactivates cytokinins, which plays important roles in plant growth and cell proliferation (Zheng et al., 2023). Similarly, enhanced grain yield was observed by deleting a target site of the transcription factor An-1 in the cis-regulatory region of the *Ideal Plant Architecture 1* (*IPA1*) gene (Song et al., 2022).

**TABLE 2:** CRISPR/Cas9-mediated gene editing in Rice.

Sr No.	Targeted genes	Role in plant	Knockout effects on plant	References
1	*sbe2b*	*sbe2b* (*Starch Branching Enzyme 2b*) is involved in starch biosynthesis, specifically in the branching of amylopectin	Reduced seed setting rate and yield	Chen et al. (2024)
2	*OsPPKL1/qGL3*	*PROTEIN PHOSPHATASE WITH KELCH-LIKE1 (OsPPKL1)* as the causal gene for the quantitative trait locus *GRAIN LENGTH3 (qGL3*) in rice	Reduced plant height, tiller numbers, and grain size	Gao et al. (2024)
	*OsWDR48*	*OsWDR48* is involved in brassinosteroid signaling		
3	*OsbZIP10*	A basic zipper family TF directly influences genes pivotal to starch synthesis	Improved rice grain quality	Jiang et al. (2024b)
4	*OsGAPDHC7*	*Glyceraldehyde-3-phosphate dehydrogenase* (*GAPDH*) encodes a major glycolytic enzyme involved in energy metabolism	Improved starch, soluble sugar, and amino acid contents	Kim et al. (2024)
5	*OsGAPC3*	Involved in the regulation of starch and proteins in rice grains	Affect the level of protein and starch content	Peng et al. (2024)
6	*OsLCG1*	The *less Chalk Grain 1* gene regulates the accumulation of amylose and amylopectin by influencing the expression of the *Wx* gene in rice	Increased Chalkiness, reduced total starch content, and increased protein and lipid content in mature seeds	Tu et al. (2024)
7	*OsSLRL2*	A transcription factor SLR1-like2 mediates the ABA-regulated amylose content of rice	Increased pre-harvest sprouting (PHS)	Wang et al. (2024c)
8	*OsCYP735A3/4, OsIPT1-10, OsLOG1, OsLOGL1-10, OsPUP1-13, OsENT1-4, OsCXX1-11*	Cytokinin metabolism-associated genes	Improved yield	Wu et al. (2024)
9	*OsLAC6*	Regulating amylose content in rice by influencing the splicing efficiency of the *Wx* gene locus	Reduced amylose content, decreased grain length, and thousand-grain weight (TGW)	Yang et al. (2024)
10	*OsMIR168a*	It targets the main RNA-induced silencing complex component AGO1 to regulate plant growth and environmental stress responses	Fast growth at the seedling stage, produced more tillers and matured early	Zhou et al. (2024a)
11	*OsHd1, OsGHD7* and *OsDTH8*	Heading date genes	Extremely early heading phenotype with low yield	Zhou et al. (2024b)
12	*OsPUB33*	*Plant U-box E3 ubiquitin ligase* (*OsPUB33*) interferes with the *OsNAC120–BG1* module to control rice grain development	Improved grain size and weight	Xie et al. (2024c)
13	*OsSBE*	It catalyzes the formation of α−1,6-glucosidic linkages of amylopectin during starch biosynthesis	Improved resistant starch levels up to 15%	Biswas et al. (2023)
	*OsCpSRP43, OsCpSRP54a,* and *OsCpSRP54b*	Play an important role in the chloroplast signal recognition particle (CpSRP) pathway	Increased Photosynthesis	Caddell et al. (2023)
14	*OsNAC24*	Transcriptional activator of starch-synthesis enzyme-coding genes	Improved starch synthesis in rice endosperm	Jin et al. (2023)
15	*OsMADS17*	*OsMADS17* encodes TF that regulates grain yield by controlling multiple genes associated with grain number and grain weight	Increase in both grain number and grain weight	Li et al. (2023c)
	*OsAP2-39*	*OsAP2-39* regulates the yield-related network and interacts with *OsMADS17*	Improved grain weight and yield	
16	*OsHHO3*	A transcriptional repressor of three *AMMONIUM TRANSPORTER1* genes	Improves nitrogen use efficiency	Liu et al. (2023a)
17	*OsMKK3*	Associated with the mitogen-activated protein kinase signaling pathway	Decreases grain length	Qing et al. (2023)
18	*OsFLO2*	A regulatory protein that controls the biosynthesis of seed storage substances	Low amylose content	Song et al. (2023)
19	*OsIPCS*	Involved in the synthesis of inositolphosphorylceramide, determine the plant architecture and influence physiological traits	Decreased growth attributes and reduced the ceramide and glucosylceramide levels	Wang et al. (2023)
20	*OsSGL2*	*SGL2* is a specific grain width regulator	Decreased grain width	Xiong et al. (2023)
	*GW8*	*GW8* is a positive regulator of grain width	Reduction in overall plant height and grain width	
	*WOX11*	*WOX11* (*Wuschel-related homeobox 11*) is involved in root and shoot development	Decreased grain width	
21	*OsAAP11*	Amino-acid-transporter-encoding gene	Increased viscosity during the cooking process, enhanced the eating and cooking quality	Yang et al. (2023)
22	*OsWx* and *OsBADH2*	*The Waxy* gene has a role in seed amylose synthesis and the *BADH2* gene has an anti-role in the synthesis of 2-acetyl-1-pyrroline (2-AP)	Creation of Two-Line Fragrant Glutinous Hybrid Rice	Zhang et al. (2023a)
23	*OsNDF6* and *OsNDHU*	Involved in the electron transport chain in the chloroplast	Decreased cyclic electron flow	Zhang et al. (2023c)
24	*OsCKX*	Plays an important role in plant growth and cell proliferation	Changed plant height, seed appearance quality and starch composition	Zheng et al. (2023)
25	*OsGLUA/B*	It encodes Glutelins: the major storage proteins in rice grains	Low glutelin content rice	Chen et al. (2022c)
26	*OsKRN2*	Encodes WD40 protein and determines kernel row number by controlling the secondary panicle branches	8% increase in grain yield by enhancing secondary panicle branches	Chen et al. (2022b)
27	*OsABA8ox*	Encodes ABA8 hydroxylase- involves ABA catabolism	Improved pre-harvest spouting resistance and enhanced seed dormancy	Fu et al. (2022)
28	*OsBADH2*	*BADH2* gene has an anti-role in the synthesis of 2-acetyl-1-pyrroline (2-AP)	Aromatic rice	Hui et al. (2022)
29	*miR166-RDD1*	Plays a role in the uptake and accumulation of various nutrient ions	Decreased grain chalkiness	Iwamoto, (2022)
30	*OsSSSII-1/2/3*	*Soluble starch synthase* (SSS) genes	High amylose content in the seeds of up to 63%	Jameel et al. (2022)
31	*OsPMEI12*	Involved in Growth, Cell Wall Development, and Response to Phytohormone and Heavy Metal Stress	Enhanced plant growth and development at a mature stage	Li et al. (2022c)
32	*OsWXB*	Plays a major role in seed amylose synthesis	Increases Grain Amylose Content	Liu et al. (2022b)
33	*OsSBE3*	*Starch Branching Enzyme 3* involved in the synthesis of amylopectin	Increase in the length of amylopectin chains, enhanced starch digestibility, and cooking quality	Shim et al. (2022)
34	*OsIPA1*	*Ideal Plant Architecture 1* gene	Enhanced grain yield	Song et al. (2022)
35	*OsGS2/GRF4*	Encodes growth-regulating factor 4 (*OsGRF4*) that regulates multiple agronomic traits	Increased rice grain size and yield	Wang et al. (2022a)
36	*OsPUB43*	U-box E3 ubiquitin ligase	Improves Grain Length and Weight	Wu et al. (2022)
37	*OsGW2*	Role in grain width and grain weight	Enhanced accumulation of iron, zinc, potassium, calcium, and phosphorous in endosperm and thick aleurone layer with higher protein content	Achary and Reddy, (2021)
38	*OsPHYC*	Function in regulating flowering time (photomorphogenesis)	Shorten the heading date	Li et al. (2021a)
39	*OsSPL16* and *GW8*	Encodes a promoter binding protein that promotes cell division and increases grain weight	Improves grain yield	Usman et al. (2021)
40	*OsHXK1*	*HXK*-phosphorylated sugars have a role in the regulation of photosynthesis-related gene expression	Improving the rice photosynthetic efficiency and yield	Zheng et al. (2021b)
41	*GS3*	Encodes a protein that restrains the cell division of spikelet hull leading to shorter grain	Slenderical grain with a lower chalkiness percentage	Yuyu et al. (2020)
42	*GL3.1*	Encodes an enzyme that controls grain size by dephosphorylating cell cycle-related protein Cyclin-T1,3 and inhibits the cell proliferation in hull	Larger grain with higher chalkiness percentage	
43	*OsDSG1*	UBA pathway and regulation of biochemical reactions in rice	Enhanced salt tolerance in rice	Ly et al. (2024)
44	*OsLCD*	Role in cadmium distribution and transport into the rice grain	Reduction in cadmium accumulation	Chen et al. (2023)
45	*OsRR22*	Involves in both cytokinin signal transduction and metabolism	Enhance rice salinity tolerance	Sheng et al. (2023)
46	*OsLKP2*	*lov kelch repeat protein 2*- the negative regulator of cuticular wax synthesis	Increased leaf size, improves the tolerance against drought	Shim et al. (2023)
47	*OsEPSPS*	Encodes the *enzyme 5-enolpyruvylshikimate 3-phosphate synthase (EPSPS),* which is crucial for the shikimic acid pathway	Developed glyphosate-resistant rice	Sony et al. (2023)
48	*OsHPPD 30 UTR*	Involve in electron chain transport mechanism	Enhanced resistance to HPPD inhibiting herbicides	Wu et al. (2023)
49	*OsALS*	Encodes an enzyme acetolactate synthase, which plays a crucial role in the biosynthesis of branched-chain amino acids	Resistance to the herbicide bispyribac-sodium (BS)	Zafar et al. (2023)
50	*OsbHLH024*	Coincide high antioxidant activities with less ROS and stabilized levels of MDA	Enhanced salt tolerance	Alam et al. (2022)
51	*OsMADS26*	An upstream regulator of stress-associated genes	Enhanced drought tolerance	Anjala and Augustine, (2022)
52	*OsPIN9*	Role in auxin efflux carrier	Chilling tolerance	Xu et al. (2022)
53	*OsPQT3*	Encodes E3 ubiquitin ligase, significantly enhances resistance to abiotic stresses	Enhanced resistance to abiotic stresses and increases grain yield	Alfatih et al. (2020)
54	*OsERA1*	Regulates ABA signaling and the dehydration response	Enhanced drought tolerance	Ogata et al. (2020)
55	*OsPUB67*	Encode U-box E3 ubiquitin ligase significantly induced by drought, salt, cold, JA, and ABA	Reduced tolerance to drought	Qin et al. (2020)
56	*OsDST*	Regulates signal transduction pathways of stomatal closure	Enhanced leaf water retention ability lower stomatal density and under drought	Santosh et al. (2020)
57	*OsPYL9*	Involves in ABA and MDA signalling	Enhance grain yield under drought	Usman et al. (2020)
58	*OsLOGL5*	Conserved 25 amino acid sequences at the C-terminal of rice cytokinin-activation enzyme-like gene	Increased grain yield under abiotic conditions	Wang et al. (2020b)
59	*OsmiR535*	*OsmiR535* in response to NaCl, Polyethylene glycol, ABA, and dehydration stresses	Increased tolerance against abiotic stresses	Yue et al. (2020)
60	*OsNAC006*	Response to stimuli, oxidoreductase activity, cofactor binding, and membrane-related pathways	Drought and heat tolerance	Wang et al. (2020a)
61	*OsPIN5bOsGS3*, and *OsMYB30*	*OsPIN5b* is an auxin carrier and has important functions in auxin balance and transport, *GS3* participates in the grain size regulatory network, and *MYB30* is cold stress related gene	Improvement of Rice Yield and Cold Tolerance	Zeng et al. (2020)
62	*OsSRL1/2*	Putative glycosylphosphatidylinositol-anchored protein	Drought tolerance (higher grain filling under stress)	Liao et al. (2019)
63	*OsNRAMP5*	*NRAMP5* is the major transporter for Cadmium and manganese uptake in rice	Reduction in cadmium accumulation	Yang et al. (2019)
64	*OsRR22*	Involve in CK signal transduction and metabolism	Enhance rice salinity tolerance	Zhang et al. (2019a)
65	*OsLRR2*	A *leucine-rich repeat protein* gene involved in immunity, stress responses, and developmental regulation	Reduced BPH (Brown planthopper) infestation and enhanced natural biological control by attracting natural enemies	Kuai et al. (2024)
66	*OsNAC*	Involves in growth, development, and stress responses	Enhanced innate immunity	Son et al. (2024)
67	*OsBSR-d1*	Negative transcription factor involves broad-spectrum resistance to rice blast	Enhances the blast resistance	Zhang et al. (2024)
68	*OsHRC*	Histidine-rich calcium-binding protein gene	Improved rice blast resistance	Ding et al. (2023)
69	*OsHPP04*	A copper metallochaperone heavy metal-associated plant protein involves in different biological processes	Enhanced resistance to rice root-knot nematode	Huang et al. (2023)
70	*OsS5H*	Salicylic acid 5-hydroxylase activity, converting SA into 2,5-dihydroxybenzoic acid (2,5-DHBA)	Broad-spectrum disease resistance	Li et al. (2023b)
71	*OsV-ATPase d*	Involves in proton translocation across membranes	Increased resistance against Southern rice black-streaked dwarf virus (SRBSDV), but it decreased resistance against Rice stripe virus (RSV) in rice	Lu et al. (2023)
72	*OsCPK18/OsCPK4*	Role in Rice Immunity	Enhanced disease resistance and yield in rice	Li et al. (2022a)
73	*OsDjA2* and *OsERF104*	Encodes a chaperone protein and APETELA2/ethylene response factor, respectively	Resistance to *Pyricularia oryzae*	Távora et al. (2022)
74	*OsXa13*	Involves in pollen development and shows recessive resistance to bacterial blight	Transgene-free bacterial blight-resistant rice with retained fertility	Li et al. (2020a)
75	*OsPFT1*	*Phytochrome and Flowering Time 1*	Sheath blight resistance	Shah et al. (2019)

Rice quality improvement is crucial because rice passes through various downstream processes, such as dehydration, milling, removal of bran, cleaning, and cooking after harvesting, and each process directly or indirectly decreases the nutrient content of rice. For instance, cleaning alone can reduce vitamin levels by 25%–60%, potassium by 20%–40%, and proteins by 3%–7% in rice (Müller et al., 2022). Combining this with the effect of climate change, the rice produced would not be useful. Therefore, biofortification is the only method that can sustain rice nutrients. Carotenoid-enriched marker-free rice has been developed by inserting two carotenoid biosynthetic genes, *SSU-CRTI* and *ZmPSY30*, using the CRISPR/Cas9 approach (Dong et al., 2020). Achary and Reddy produced a rice variety with an enhanced accumulation of iron, zinc, potassium, calcium, and phosphorous in endosperm via CRISPR/Cas9-mediated editing of *GW2* (Grain width and grain weight) locus (Achary and Reddy, 2021). Moreover, the aleurone layer gained thickness with an enhanced protein content. Another study found decreased grain chalkiness, high ammonium cation, and phosphate ion uptake, and high photosynthetic activity under high CO_2_ conditions in miR166-RDD1 knocked-out rice plants (Iwamoto, 2022).

Among abiotic stressors, salinity is a serious event that can potentially tarnish rice production. The development of salt-tolerant varieties is a lifesaving approach feasible with CRISPR/Cas9 genome editing technology. High salt concentrations can negatively affect crop production by disrupting the metabolic and physiological processes. These factors can significantly affect plant development, seed germination, and productivity (Zörb et al., 2019). Plants respond to salt stress by increasing the biosynthesis of antioxidants, osmoregulators, and phytohormones that support the plant by maintaining ion homeostasis; however, this ability works to a certain extent, and not all plants can protect themselves from high salinity (Raza et al., 2022). Similarly, drought stress drastically affects the physiology of plants by inhibiting nutrient uptake and other life-dependent activities, including photosynthesis, cell division and elongation, turgor pressure, and gene expression. Several rice genes have been identified and mutated using CRISPR/Cas9 to produce abiotic stress-tolerant plants (Table 2). For example, salt-tolerant T2 homozygous mutant rice was developed by cleaving the *OsRR22* gene in rice using Cas9 (Zhang A. et al., 2019). Knockout of *OsRR22* improved the performance of rice plants under high-salt conditions (0.75%), with no side effects on grain size, yield, or plant biomass. The same strategy was used by Han et al. to develop novel rice germplasm at the seedling stage (Han et al., 2022). In another study, the drought and salt tolerance (*OsDST*) gene was edited using CRISPR/Cas9 to produce the indica mega rice cultivar MTU1010 with enhanced tolerance (Santosh et al., 2020). In rice, the transcription factor *OsMADS26* plays a negative role, and mutations in this gene enhance drought tolerance (Anjala and Augustine, 2022). Ogata et al. characterized the *Enhanced Response to ABA1* (*ERA1*) gene in rice using CRISPR/Cas9-mediated editing and found frameshift mutations in mutants with increased primary root growth, high sensitivity to abscisic acid stress, and increased drought stress tolerance (Ogata et al., 2020).

The effect of climate change on disease susceptibility in rice poses a major threat to rice productivity. Climate change-induced pest and microbial emergence can annihilate agriculture, thereby substantially threatening food security. Fortunately, this can be mitigated using gene-editing applications in crops. Various studies have been published on the production of pests and microorganisms that cause disease tolerance in crops (Liu M. et al., 2024; Oliva et al., 2019; Zhou et al., 2022). Table 2 shows some examples of CRISPR/Cas9-mediated development of disease-resistant plants. However, the current work is insufficient in comparison to the upcoming consequences of climate change because this change can also increase the potential of insects by providing them with favorable conditions for their growth and development. For example, fruit flies flourished more when the temperature increased from 20°C to 35 °C in certain mango varieties. This phenomenon is observed in all species worldwide. In particular, insects in temperate regions have become more active, whereas populations of tropical insects may decline or migrate (Bhattacharjee et al., 2022). Therefore, genetically edited plants may be a powerful solution for overcoming the effects of climate change on agriculture. Several studies have been conducted to develop rice resistance to various fungal and bacterial pathogens. *Xa13* is involved in pollen development and exhibits recessive resistance to bacterial blight. Studies have shown that the complete loss of function of the coding region of this gene can lead to sterility; therefore, CRISPR-assisted modification was performed in the *Xa13* promoter region to produce transgene-free bacterial blight-resistant rice with retained fertility (Li C. et al., 2020). Another study targeted three *salicylic acid 5-hydroxylase* (*OsS5H*) genes (*BSR-D1*, *PI21*, and *ERF922*) in rice and found resistance to both rice blast and bacterial blight (Zhou et al., 2022). Recently, rice with enhanced immunity was produced by editing the NAC transcription factor gene in rice via CRISPR/Cas9 approach (Son et al., 2024). Furthermore, broad-spectrum disease resistance was achieved by editing three *salicylic acid hydroxylase* (*OsS5H*) genes in rice (Li X. et al., 2023).

### 3.3 Maize: improvement of traits via the CRISPR/Cas9 approach

Maize (*Zea mays*) is the most important cereal crop in the world, with the highest production after rice and wheat, and fulfills the needs of human food, animal feed, and biofuels (Chávez-Arias et al., 2021). Maize is sensitive to heat stress during seed germination and vegetative growth. It significantly affects maize plant germination and seedling emergence. Heat stress causes the formation of abscisic acid and affects the activity of enzymes responsible for breaking down starch (Chandra et al., 2023). Additionally, it inhibits the synthesis of proteins in the embryo, which reduces the germination of maize seeds at over 37°C, resulting in a decrease in plant density (Buriro et al., 2011). Increased oxidative stress, altered membrane permeability, decreased stomatal conductance, and other signs occur regularly in plants under heat stress. The rate of photosynthesis was negatively affected by a reduction in stomatal conductance. Moreover, it induces the production of reactive oxygen species and causes oxidative stress (Soengas et al., 2018; Zandalinas et al., 2017). Heat stress negatively affects the number of florets, silk number, fertilization, filling, development, and final grain yield during flowering (Lizaso et al., 2018). Recently, the continuous span of heat waves has affected maize yield and productivity. They alter the morphology, physiology, genomic expression, and biochemical metabolism of crops. In response to these alterations, plants activate tolerance mechanisms via heat shock transcription factors and proteins essential for reducing and preventing heat-related damage (Li and Zhang, 2022d). Heat stress affects the integrity of the plasma membrane and the accumulation of reactive oxygen species (Dogra and Kim, 2020). Moreover, proteins are misfolded or unfolded, which disrupts cell metabolism and physiology and eventually leads to cell death. The development of climate resilience in maize is urgently needed because heat-induced decline is high in this crop (Chandra et al., 2023).

Currently, a decrease in yield resulting from drought stress is estimated to affect over 20% of the annual maize area, and at high temperatures, an average of 7.4% is lost for every 1°C increase (Boyer et al., 2013; Malenica et al., 2021). Notably, Brazil, the third largest producer of maize worldwide, showed a decrease in yield in the 2015–16 and 2020–21 growing seasons of approximately 18 and 23 Mt, correspondingly to losses of approximately 21% compared to the 2014–15 and 2019–20 seasons (Lopes Filho et al., 2023). These crop failures occurred in years marked by extreme drought conditions, resulting in diminished yields in many of the largest producer geographies. Similarly, crop yield reductions and spiking prices were affected by the 2012 drought in the US (the world’s largest producer) (Boyer et al., 2013). Therefore, it is essential to reduce the potential losses caused by the increased frequency, severity, and duration of stresses associated with global climate change by continuously developing new maize cultivars that target better genetic adaptation and using improved agricultural practices.

Abiotic stress tolerance and yield are two complex traits strongly influenced by environmental factors and linked to small-effect genetic loci. Using genomic engineering techniques to develop superior cultivars for these traits is more difficult because of such complexity, which makes it challenging to reliably evaluate the molecular mechanisms behind gene activities and measure phenotypes. Considering the few instances developed for complex traits, transgenic maize cultivars with enhanced herbicide and insect resistance have been on the market for decades (Yassitepe et al., 2021). The difficulty in applying a transgenic approach to control complex traits that are stable in multiple environments has limited the development of biotech cultivars that could be widely used (Simmons et al., 2021).

Gene editing technology has enabled an efficient and consistent way to understand the role of key genes and develop new germplasm in maize (Doll et al., 2019; Wang Y. et al., 2022).

Potential putative functions of numerous genes involved in maize development programs and stress responses have been investigated thoroughly using CRISPR/Cas9 technology (Wang Y. et al., 2022). CRISPR has been widely used to enhance numerous agronomic traits in maize, yield, nutrition, improved pollen characteristics, drought tolerance, and disease resistance (Jiang L. et al., 2024; Kaul et al., 2024; Lv et al., 2024; Wang G. et al., 2024; Xie Z. et al., 2024). *ZmGDIɑ* was specifically edited using CRISPR/Cas9 to significantly increase maize resistance to the maize rough dwarf virus without negative agronomic effects (Liu Y. et al., 2023). *ZmCOIɑ* interacts inversely with *ZmJAZ15* to alter maize immunity against *Gibberella* stalk rot (GSR, a teleomorph of *Gibberella zeae*), and further downregulation of *ZmCOIɑ* can increase maize resistance to GSR (Ma et al., 2021). *MMS21* maintains the activity and integrity of the maize genome, resulting in improved root and vegetative growth, pollen germination, and seed development (Zhang et al., 2021b). *ZmCLCg* positively regulates sodium chloride stress and chloride transport in maize as a stress response (Luo et al., 2021). *ZmSRL5* is essential for sustaining cuticular wax structure and drought tolerance in maize (Pan Y. et al., 2020).

Maize yield is severely affected by several environmental factors, including drought, high temperatures, floods, and unsuitable soil conditions. The breeding of stress-tolerant variants has shown great potential when applying genome editing tools compared with conventional breeding methods (Chávez-Arias et al., 2021; Chennakesavulu et al., 2021; Prasanna et al., 2021). The development of maize lines with high stalk strength has become considerably important to breeders for maintaining high and constant production, as stalk lodging caused by different environmental factors poses a significant threat to maize quality and production. *STIFF1* is a negative regulator of maize stalk length, its altered allele with a 2bp deletion caused a frameshift and an early slowdown translation, conferring CRISPR-edited plants with a stronger stalk, which contributed to high-density planting and avoided stalk lodging (Armarego-Marriott, 2020). Furthermore, *ZmGA20OX3* has been modified to develop semi-dwarf maize plants using CRISPR/Cas9 technology, which may be useful for developing a novel genotype that is more resilient to lodging and suitable for high-density planting (Liu Y. et al., 2023). For drought tolerance, precisely editing the promoter sequence of *ARGOS8* leads to an increase in its expression and enhances maize grain yield under drought stress (Shi et al., 2017). By inserting the *GOS2* promoter from maize plants at the 5ʹ untranslated regions that remain of the *ARGOS8* gene’s native promoter, which acts as a negative regulator of ethylene responses (Zafar et al., 2020). Targeted alteration of the native maize promoter using CRISPR/Cas9 enhanced *ARGOS8* expression and improved grain production under drought conditions. In addition, CRISPR/Cas9 has been used to target *Slagamous-Like 6* (*SIAGL6*) to achieve heat tolerance (Doll et al., 2019). Therefore, developing new germplasm sources for breeding stress-tolerant maize has been achieved using CRISPR/Cas9 technology. Table 3 illustrates several improvements in the agronomic traits of maize using CRISPR/Cas9 gene editing.

**TABLE 3:** CRISPR/Cas9-mediated gene editing in Maize.

Sr No.	Targeted genes	Role in plant	Knockout effects on plant	References
1	*ZmTKPR1-1/2*	*Tetraketide α-pyrone reductases* gene forms an important precursor of sporopollenin	Male sterility with delayed tapetum degradation and defective pollen exine and anther cuticles	An et al. (2024)
2	*ZmWUS1-B*	Embryogenesis-related gene *WUSHEL* regulates the stem cell population in inflorescence meristems	Affect inflorescence meristem	Chen et al. (2021)
3	*ZmMSH7*	DNA repair-related genes confer natural variation in maize pollen fertility	Boosts grain yield	Jiang et al. (2024a)
4	*ZmBON1/3*	Plasma membrane-associated copine proteins are critical components of Brassinosteroids signaling	Produce dwarf morphology	Jing et al. (2024b)
5	*ZmEPSPS, GATIPS-mZmEPSPS*	Shikimic acid pathway gene	High yield and enhanced glyphosate resistance	Kaul et al. (2024)
6	*ZmMPK6*	Mitogen-activated protein kinase 6 role in key signaling enzymes involved in stress responses, cell division, metabolism, and plant growth	Reduction in kernel weight	Li et al. (2024b)
7	*ZmADF1*	Actin-binding protein	Greater pollen viability	Lv et al. (2024)
8	*ARFTF17*	It encodes a protein that inhibits MYB40, a transcription factor with the dual functions of repressing *PIN1* expression and transactivating genes for flavonoid biosynthesis	Reduces IAA content in the seed pericarp, creating a flint-like kernel phenotype	Wang et al. (2024b)
9	*ZmARF1*	Auxin response factors (ARFs) play crucial roles in root development via auxin signaling mediated by genetic pathways	Shorter primary roots, fewer root tip number, and lower root volume and surface area	Yan et al. (2023)
10	*ZmRA2* and *ZmTSH4*	*RA2* is a RAMOSA pathway member that generates highly branched tassel. *TSH4* represses lateral organ growth and also affects phyllotaxy, axillary meristem initiation, and meristem determinacy within the floral phase	Increased tassel branch number	Xie et al. (2024b)
11	*ZmNDF6* and *ZmNDHU*	*NDF6* is an integral part and a trans-membrane subunit of the NDH complex, and *NDHU* is a chloroplast-specific subunit located close to the electron donor ferredoxin binding site	Retarded growth, low leaf chlorophyll contents	Zhang et al. (2023b)
12	*Zm00001d016075*	Negatively modulating kernel row number	Increased kernel row number and grain yield	An et al. (2022)
13	*ZmKRN2*	Encodes WD40 protein and determines kernel row number	10% increase in grain yield	Chen et al. (2022a)
14	*ZmPAT7*	Phosphate transporter	Increased tassel branch number	Guan et al. (2022)
15	*ZmDFR1/2* and *ZmACOS5-1/2*	Regulating anther and pollen development	Defective anther and pollen, male fertility	Liu et al. (2022c)
16	*ZmCOI2a/b*	Receptor of jasmonate signal	Defective anther, male sterility	Qi et al. (2022)
17	*ZmSPL12*	SPL transcription factor	Increased height and ear height	Zhao et al. (2023)
18	*ZmNL4*	Regulating cell division	Reduced leaf width	Gao et al. (2021)
19	*ZmThx20 GT-2*	Trihelix transcription factor	Shrunken kernels	Li et al. (2021c)
20	*ZmAGAP*	Arf GTPase-activating protein	Dwarfed plant, smaller ear, and small leaf	Jia et al. (2020)
21	*ZmCLE7, ZmFCP1, ZmCLE1E5*	CLE peptide ligands	Increased multiple grain-yield-related traits	Liu et al. (2021)
22	*YIGE1*	Regulating ear length by affecting pistillate floret number	Decreased inflorescence meristem size and ear length	Luo et al. (2021)
23	*ZmACO2*	Ethylene biosynthesis	Enhanced ear length, kernel number, and grain yield	Ning et al. (2021)
24	*ZmBADH2a/b*	2-acetyl-1-pyrroline biosynthesis	Aromatic corn	Wang et al. (2022b)
25	*ZmCEP1*	Peptide hormones	Increased plant height, kernel size, and weight	Xu et al. (2021)
26	*MSCA1, ZmGRX2/5*	Modifying the redox state and the activity of their target proteins	Suppressed meristem, reduced height	Yang et al. (2021)
27	*MMS21*	SUMO ligase	Short root, abnormal seed	Zhang et al. (2021a)
28	*ZmACS7*	Ethylene biosynthesis	Increase in plant height, ear height, above internode number, and leaf angle	Li et al. (2020b)
29	*ZmANT1*	AP2 transcription factor	Reduced growth rate and grain yield	Liu et al. (2020)
30	*STIFF1*	F-box domain protein	Stronger stalk strength	Zhang et al. (2020)
31	*ZmNRPC2*	Second-largest subunit of RNA polymerase III	Reduced kernel size	Zhao et al. (2020)
32	*ZmADF5*	Member of the actin-depolymerizing factor (ADF) family, tightly linked with a consensus drought-tolerant quantitative trait locus	Decreased drought tolerance	Liu et al. (2024a)
33	*ZmPP2C15*	The protein phosphatase is involved in plant growth and development and various signaling pathways	Severe leaf dryness, curling, and wilting under drought stress	Pang et al. (2024)
34	*ZmEREB24*	Drought stress-responsive *AP2* gene	Drought sensitivity	Ren et al. (2024)
35	*ZmHDT103*	Maize nutrition and reproductive development	Drought Stress Tolerance	Wang et al. (2024e)
36	*ZmGA20ox3*	GA biosynthesis	Improves plant architecture and drought tolerance	Liu et al. (2023b)
37	*ZmbHLH32* and *ZmIAA9*	bHLH transcription factor for *ZmIAA9* gene a member of the maize *Aux/IAA* gene family	Increased sensitivity to salt stress, decreased ROS detoxification	Yan et al. (2023)
38	*ZmCLCg*	Chloride transport	Reduced salt tolerance	Luo et al. (2021)
39	*ZmSRL5*	Cuticular wax related gene	Reduced drought tolerance	Pan et al. (2020b)
40	*ZmADT2*	Arogenate dehydratase- downstream enzymes of chorismite mutase	Increased susceptibility to *Ustilago maydis* fungus	Ren et al. (2024)
41	*ZmPDRP1/2*	Involves in C4 photosynthesis of maize	Resistance to potyvirus sugarcane mosaic virus (SCMV)	Xie et al. (2024a)
42	*ZmPR5L* and *ZmRBOH4*	Cell-wall-associated receptor kinase-like protein	Reduced plant height and increased gray leaf spot susceptibility	Zhong et al. (2024b)
43	*ZmGDIa*	Vesicle membrane trafficking	Disease resistance	Liu et al. (2022a)
44	*ZmCOI1a, ZmJAZ15*	Jasmonate signaling components	Disease resistance	Ma et al. (2021)

## 4 Bibliometric analysis

A bibliometric analysis was conducted to examine global trends in CRISPR/Cas9 research, focusing on cereal crops and their applications for stress tolerance and yield improvement. The search used terms like “CRISPR,” “Genome editing,” “Gene editing,” and “Gene silencing,” along with crop-related terms such as “Cereal crops” and stress-related topics like “Abiotic stress,” “Biotic stress,” and “Drought tolerance.” The dataset, initially containing 1,232 articles from 2019 to 2025, was narrowed to 597 after excluding reviews, book chapters, and non-English publications. The citation analysis and other visualizations were conducted using Web of Science (WoS) and VOSviewer.

**FIGURE 4:**
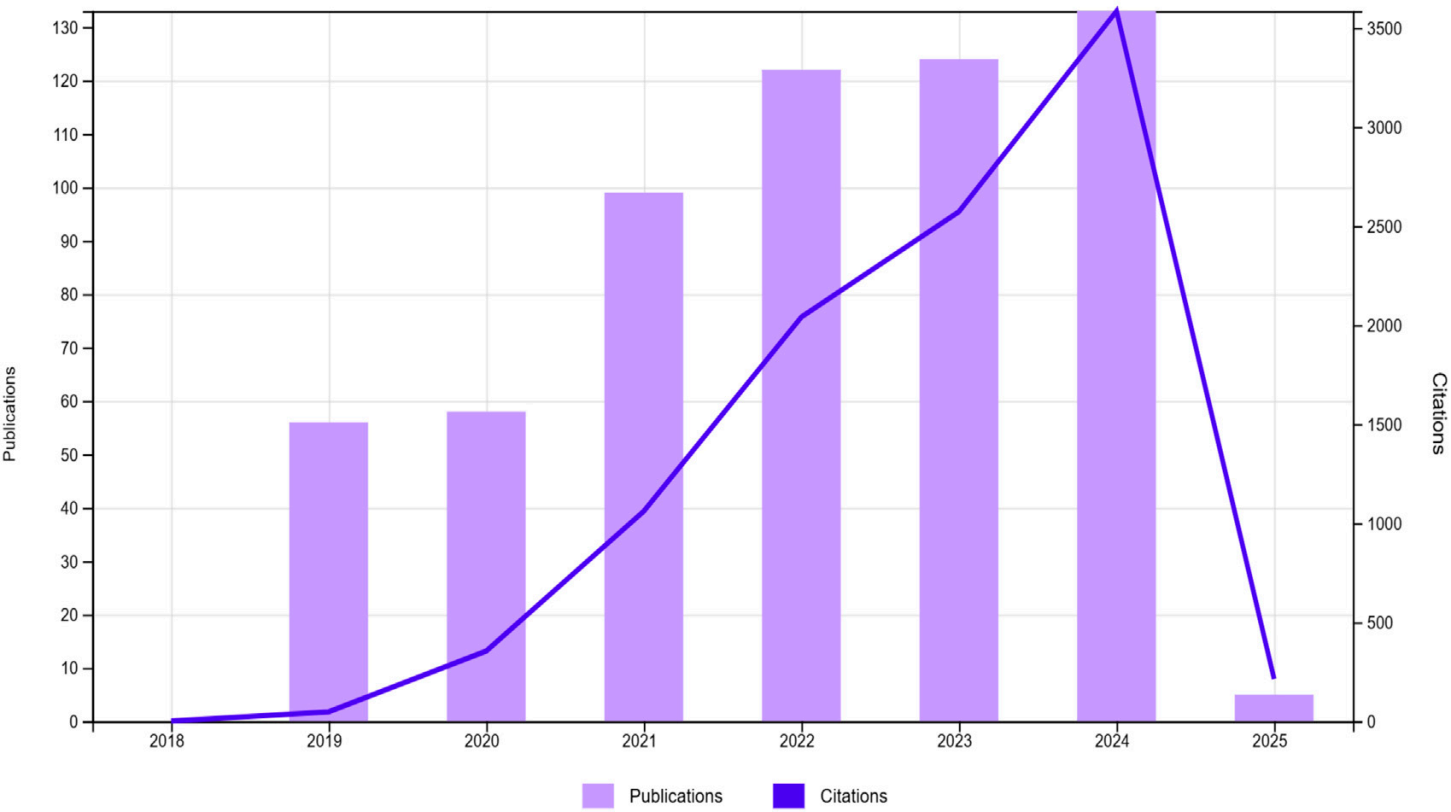
Annual publication trends from 2019 to 2025, showing the number of papers published each: The graph illustrates a surge in publications and citations from 2019 to 2025 in the area of CRISPR/Cas9 for enhancing crop stress tolerance and yield Created with BioRender.

**FIGURE 5:**
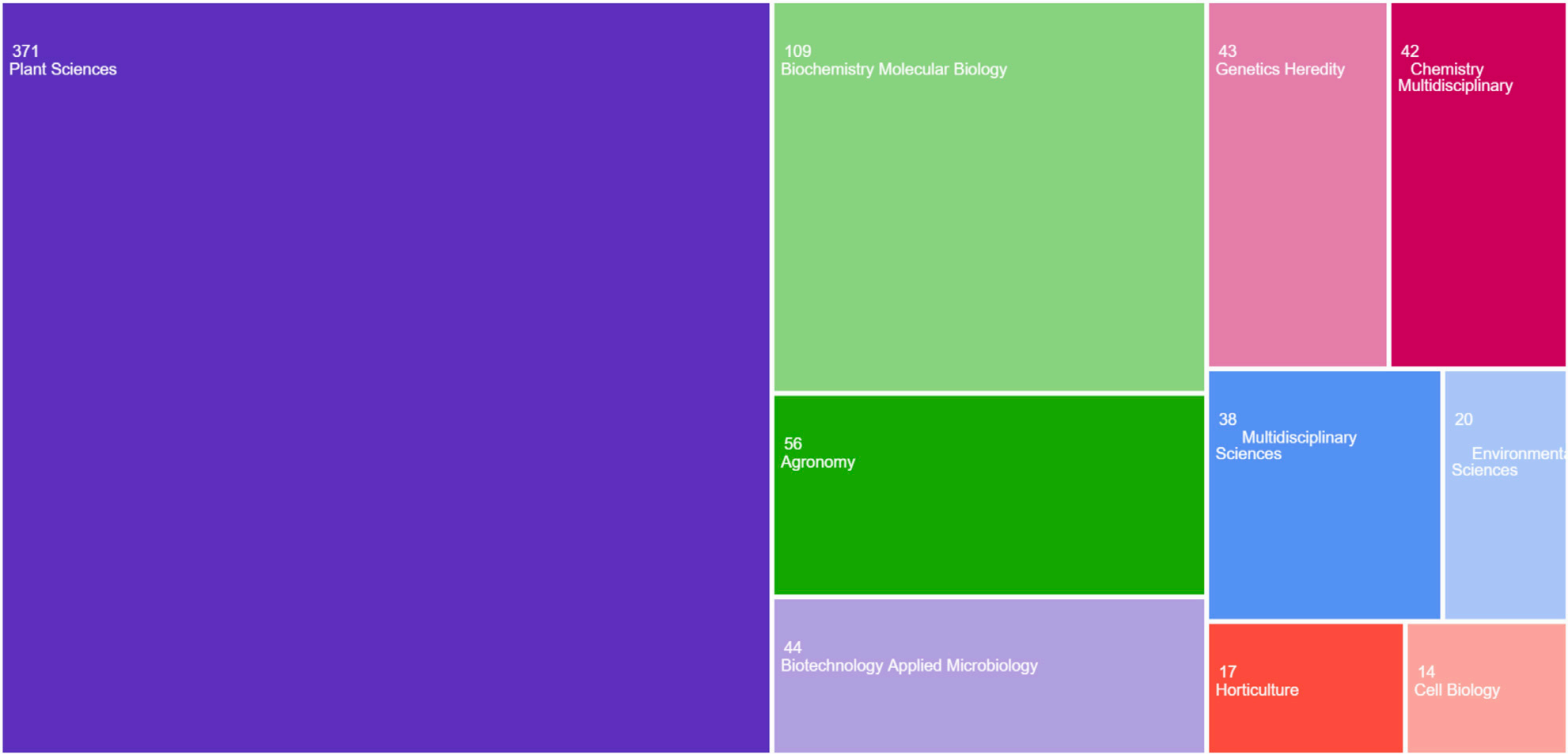
The tree map illustrates research across different subject areas: This map focuses on research across different subject areas, with Plant Sciences comprising the largest share, followed by biochemistry, molecular biology and agronomy Created with BioRender.

According to the database, increasing trend in publications and citations from 2019 to 2025 can be seen, demonstrating a significant increase in scholarly interest in CRISPR/Cas9 for enhancing crop stress tolerance and yield (Figure 4). After 2021, both publications and citations remained steady, suggesting the field’s maturation or a shift in research focus, with sustained interest continuing in the subsequent years (Francis et al., 2024). The tree map in Figure 5 organizes research across different subject areas, with Plant Sciences comprising the largest share, followed by biochemistry, molecular biology and agronomy. This distribution emphasizes the central focus on plant traits, particularly in cereals, while also highlighting the broader applications of CRISPR/Cas9 in fields like biochemistry and biotechnology. Smaller categories, such as Horticulture and Environmental Sciences, reflect the interdisciplinary nature of genome editing research (AlRyalat et al., 2019).

**FIGURE 6:**
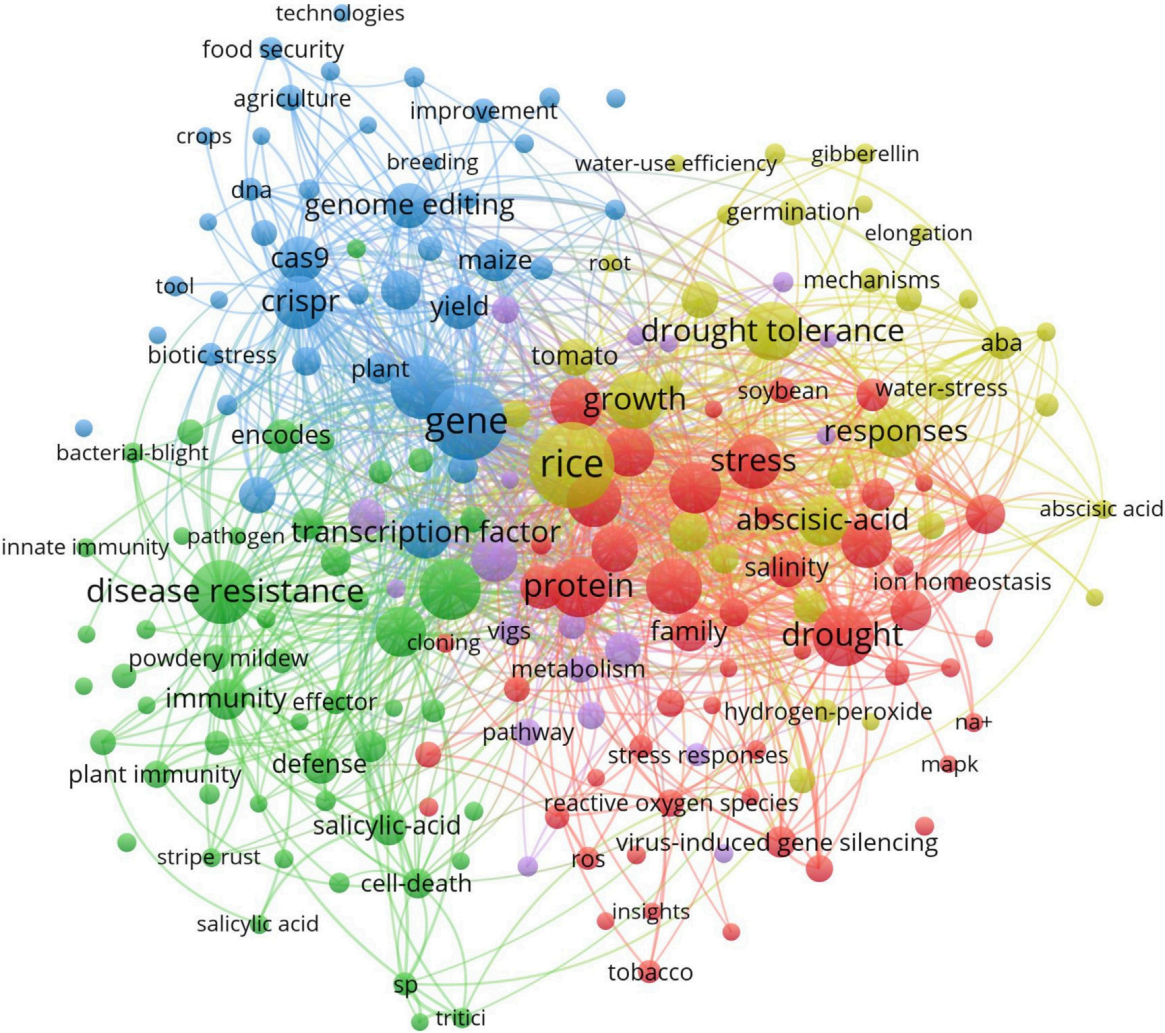
Network visualization of keyword co-occurrence related to CRISPR/Cas9 research in cereals: The central nodes in the figure illustrates the relationship between CRISPR and genome editing specially in the research areas such as drought tolerance, yield improvement, and stress resistance, with a primary focus on rice. Visualization uses different colors to distinguish between research areas, with red highlighting drought tolerance, green representing stress resistance, and blue focusing on gene editing Created with BioRender.

The network visualization in Figure 6 further shows the relationships between key research themes. The central nodes CRISPR and genome editing are closely connected to other significant areas such as drought tolerance, yield improvement, and stress resistance, with a primary focus on rice, a key cereal crop. This network highlights the broad applications of CRISPR/Cas9 in enhancing abiotic stress tolerance and improving crop yield. The strong links between these themes suggest that improving crop resilience is a major goal in CRISPR/Cas9 research (Altaf et al., 2024).

**FIGURE 7:**
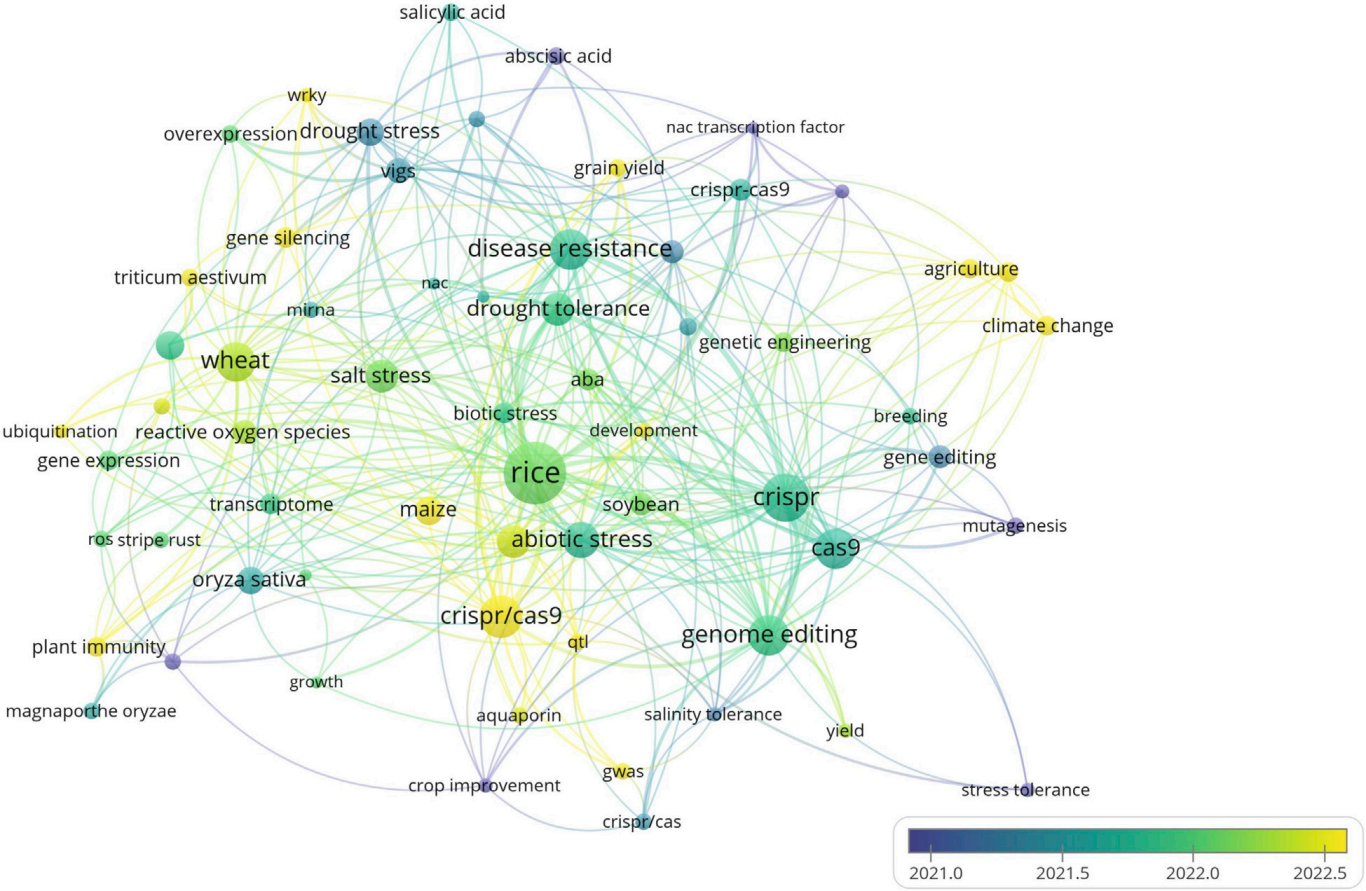
The overlay visualization of research trends on CRISPR/Cas9 over time: The figure shows the research trends over time, especially focusing on drought tolerance, salinity tolerance, and abiotic stress. Since 2021, the research has increasingly focused on these topics, particularly in cereal crops like rice, wheat, and maize Created with BioRender.

The overlay visualization, tracks how research trends have changed over time, especially focusing on drought tolerance, salinity tolerance, and abiotic stress (Figure 7). From 2021 onward, the research has increasingly focused on these topics, particularly in cereal crops like rice, wheat, and maize. This shift reflects the growing need for developing climate-resilient crops, with more research being dedicated to improving stress resistance and water use efficiency in cereals. The overlay emphasizes the growing interest in improving cereals to make them more resilient and higher yielding under extreme environmental conditions. While Figure 6 shows the connections between topics, Figure 7 illustrates the evolution of these topics over time, especially highlighting cereals as a key focus in the search for more resilient crops to face climate change (Altaf et al., 2024).

**FIGURE 8:**
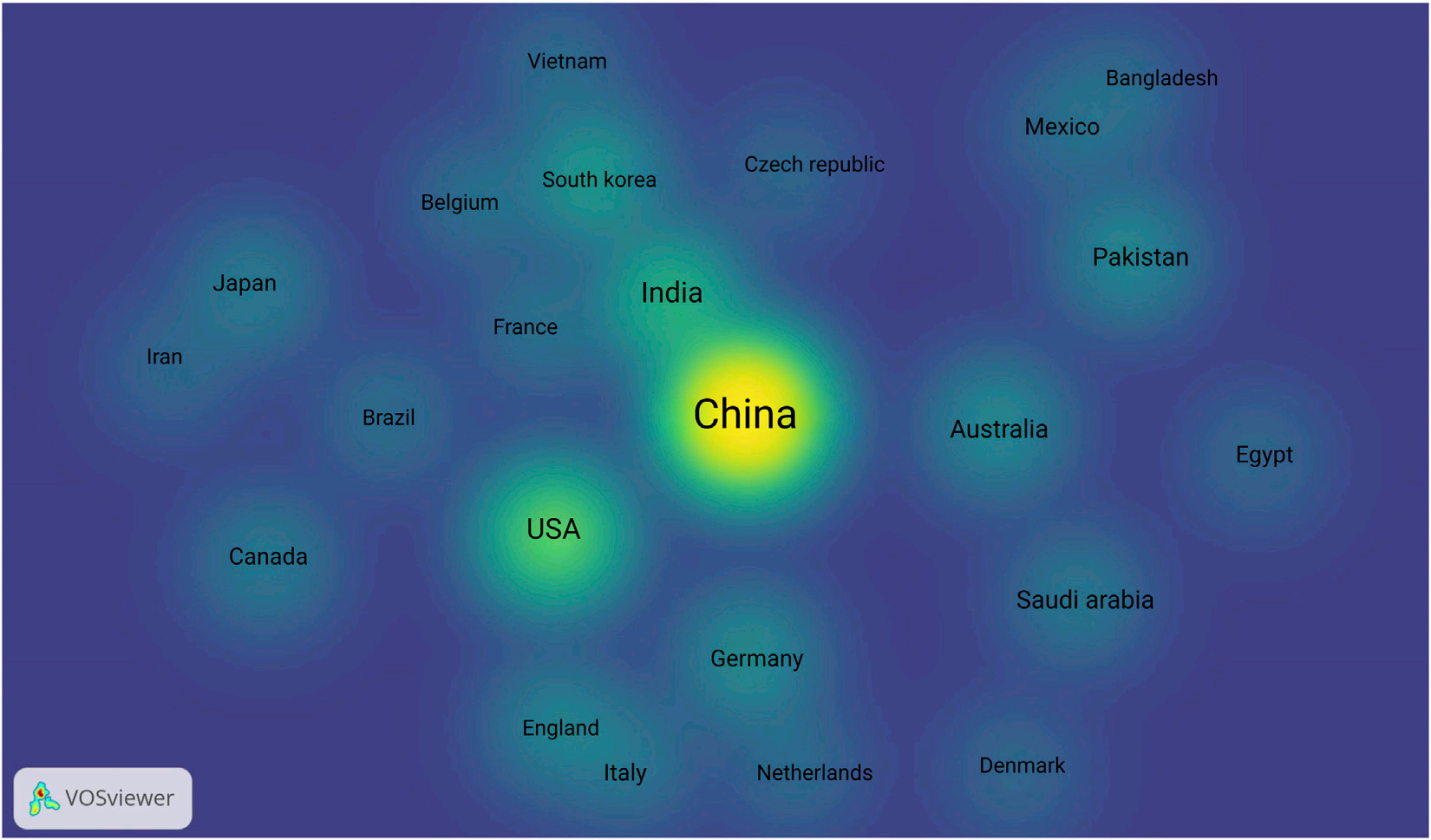
Country-wise distribution of publications on CRISPR/Cas9 research in cereals from 2019 to 2025: The heatmap reveals that countries such as China, United States, and India are at the forefront of this research, with other countries like South Korea, Brazil, and France contributing significantly Created with BioRender.

Furthermore, the country distribution map, highlights the geographic spread of research on CRISPR/Cas9 applications in cereal crops (Figure 8). The heatmap reveals that countries such as China, United States, and India are at the forefront of this research, with other countries like South Korea, Brazil, and France contributing significantly (Martins et al., 2022). However, there is a noticeable gap in regions that rely heavily on cereal crops, such as the Middle East. This suggests an opportunity for further research in these areas, particularly to address local agricultural challenges related to climate stress and water scarcity.

## 5 Limitations, advancements, and future challenges of gene editing

Gene editing has been successfully used to generate climate-resilient crops for various climatic conditions. However, several limitations are descending its overall potential remains limited (Singh et al., 2024a; Singh et al., 2024b). The major drawback of gene-editing technologies is off-target, which can cause unwanted editing of other genes, which hinders their wide applicability for crop trait improvement.

Another disadvantage of, the lack of efficient tissue culture methods for regeneration, transformation and generation of gene edited crops. The use of a *de novo* meristem induction technique can be more useful and easier for recalcitrant crop species (Maher et al., 2020). Research is being conducted to increase the transformation ability of recalcitrant varieties using advanced tools to produce climate-resilient crops.

Apart from these technical drawbacks, policymakers and regulatory authorities must take the initiative to overcome the lack of clarity regarding genome-edited crops among the population. Altogether, these factors can provide ultimate success in applying these technologies to address the impact of climate change.

Intragenic, transgenic, and cisgenic (ICT) approaches have been used to improve plant characteristics using foreign genes which leverage its applications (Karavolias et al., 2021; Klümper and Qaim, 2014; Steinwand and Ronald, 2020).

Therefore, merging genome editing with ICT approaches would be the best solution for raising climate-smart crops and this can be possible using targeted gene integration using CRISPR/Cas9 technology. This strategy has been applied to maize varieties by successfully inserting a novel promoter upstream of a gene responsible for ethylene regulation to improve drought tolerance. Moreover, the entire gene can be replaced using these approaches; for example, the replacement of the japonica *NRT1.1B* allele with the indica allele improves nitrogen use efficiency in rice (Li et al., 2018). Although these approaches have great potential, regulations regarding these technologies have decreased their feasibility. Therefore, new advancements and technical improvements are required to overcome these limitations.

For example, base editors and prime editors are second-generation CRISPR-based genome modification tools that mediate precise editing without relying on double-stranded break formation and homology direct repair. These editors are more precise in terms of single-nucleotide modification and integration (Lin et al., 2020). Base editors involve the direct conversion of a single-nucleotide base into another (A-to-G or C-to-T, and A-to-C or C-to-G), without forming double-strand breaks, introducing specific point mutations with utmost precision. Base editing has been applied to several crops including Arabidopsis, cotton, rice, tomato, maize, tobacco, and soyabean (Li X. et al., 2024; Luo et al., 2023; Wang G. et al., 2024; Wang et al., 2024f; Wei et al., 2023; Zhong D. et al., 2024). On the other hand, prime editors integrate Cas9 nickase and a reverse transcriptase, prime editing guide RNA (pegRNA) which is a combination of Cas9 sgRNA, a reverse transcriptase template, and a primer-binding site (PBS). The pegRNA guides the nCas9 to the target site, where it makes a nick in the non-target DNA strand. Then the reverse transcriptase extends the nicked strand by utilizing the reverse transcriptase template (RTT) from the pegRNA, thereby incorporating the intended modifications. With prime editors, large deletion, replacement, and inversion of larger DNA fragments can be performed in plants with high precision. Researchers have achieved DNA inversions of up to 205.4 kb in wheat plants with 51.5% by using dual prime editors. They have also been applied to edit large DNA fragments in tobacco and tomato (Zhao et al., 2025). Similarly, a recent study utilized high-efficiency prime-editing tools to knockin a 10-bp heat-shock element (HSE) into promoters of *cell-wall-invertase genes* (*CWINs*) in rice and tomato cultivars (Lou et al., 2025). These modified CRISPR tools can leverage the gene editing efficiency of manipulating chromosomes and larger DNA segments for crop improvement.

## 6 Conclusion

Gene editing could be a powerful solution for the present and future anticipation of climate change consequences. With the emergence of advanced genome editing techniques, including CRISPR/Cas9, base editing, and prime editing, various agronomic traits such as disease resistance, abiotic stress tolerance, and nutritional enhancement have emerged. Despite this, most gene editing technologies are still under laboratory research and have not yet been translated into the real world. This is due to technical limitations and restrictions imposed by regulatory authorities and policymakers. However, technological innovations are rapidly expanding owing to the ongoing efforts of public and private institutions. The potential of gene editing in offering solutions for climate change in agriculture is not overlooked, even though it is not the only solution to improve agriculture. Numerous studies show that gene editing can be used to enhance agriculture and combat climate change effects greatly. Nevertheless, as indicated by bibliometric analysis, significant research gaps remain, particularly in applying CRISPR/Cas9 to underexplored crops like rice, wheat and maize for comprehensive climate resilience.

## Glossary

*BADH2*: Betaine Aldehyde Dehydrogenase 2
BE: Base editing
**CO**_**2**_: Carbon dioxide
CRISPR/Cas: Clustered Regularly Interspaced Short Palindromic Repeats and CRISPR-Associated System
*DST*: *Drought and salt tolerance* gene
*ERA1*: *Enhanced Response to ABA1* gene
FAO: Food and Agriculture Organization
GBSS: Granule-bound starch synthase gene
GlutEnSeq: Gluten gene Enrichment and Sequencing
GM: Genetically Modified
GMOs: Genetically Modified Organisms
gRNA: Guide RNA
GSR: Gibberella stalk rot
HAG1: Histone acetyltransferase gene
HSFA6e: Heat shock transcription factor gene
ICT: Intragenic, transgenic, and cisgenic approaches
IPA: Ideal Plant Architecture 1 gene
IPK1: Inositol Pentakisphosphate 2-Kinase 1 gene
IUCN: International Union for the Conservation of Nature
MLO: Mildew resistance locus O
PAM: Protospacer Adjacent Motif
PFT1: Phytochrome and flowering time 1 gene
PINb: Puroindoline b gene
PPO: Polyphenol oxidase gene
PSY: Phytoene synthase gene
PYR1: Pyrabactin resistance 1 gene
RNAi: RNA interference
RNP: Ribonucleoprotein
S genes: Susceptible genes
S5H: Salicylic acid 5-hydroxylase genes
SBEIIa: Starch-branching enzyme (SBE) II gene
SIAGL6: Slagamous-Like 6 gene
SlMAPK 3: Mitogen-activated protein kinase gene
TALENs: Transcription activator-like effector nucleases
T-FACE: Temperature-free air CO2 enrichment systems
TFs: Transcription factors
TMT3B: Tonoplast monosaccharide transporter 3 gene
TRM: TONNEAU 1-recruiting motif
TRXH9: Thioredoxin gene
USA: United States of America
WSSMV: Wheat spindle streak mosaic virus
WYMV: Wheat yellow mosaic virus
ZFNs: Zinc finger nucleases
