# Supplementary material for: CRISPR/Cas9: a sustainable technology to enhance climate resilience in major Staple Crops
Source: Front Genome Ed. 2025 Mar 18;7:1533197. doi: 10.3389/fgeed.2025.1533197 (PMC11958969; doi:10.3389/fgeed.2025.1533197)
Supplement: Supplementary file 1 [file Table1.docx]

Supplementary Material

# References which are cited in the tables but not cited in the text.

## References in Table 1: CRISPR/Cas9-mediated gene editing in Wheat.

Biswal, A. K., Hernandez, L. R. B., Castillo, A. I. R., Debernardi, J. M., Dhugga, K. S. (2023). An efficient transformation method for genome editing of elite bread wheat cultivars. Front. Plant Sci. 14:1135047. https://doi.org/10.3389/fpls.2023.1135047

Chang, Y., Tang, H., Wang, S., Li, X., Huang, P., Zhang, J., Wang, K., Yan, Y., Ye, X. (2024). Efficient induction and rapid identification of haploid grains in tetraploid wheat by editing genes *TtMTL* and pyramiding anthocyanin markers. Front. Plant Sci. 15. https://doi.org/10.3389/fpls.2024.1346364

Chen, H., Su, Z., Tian, B., Hao, G., Trick, H. N., Bai, G. (2022a). *TaHRC* suppresses the calcium-mediated immune response and triggers wheat *Fusarium head blight* susceptibility. Plant Physiol. 190(3), 1566–1569. https://doi.org/10.1093/plphys/kiac352

Chen, Z., Ke, W., He, F., Chai, L., Cheng, X., Xu, H., Wang, X., Du, D., Zhao, Y., Chen, X. (2022d). A single nucleotide deletion in the third exon of *FT‐D1* increases the spikelet number and delays heading date in wheat (*Triticum aestivum* L.). Plant Biotechnol. J. 20, 920-933. https://doi.org/10.1111/pbi.13773

Errum, A., Rehman, N., Uzair, M., Inam, S., Ali, G. M., Khan, M. R. (2023). CRISPR/Cas9 editing of wheat *Ppd-1* gene homoeologs alters spike architecture and grain morphometric traits. Funct. Integr. Genom. 23:66. <https://doi.org/10.1007/s10142-023-00989-2>

Fan, Y., Li, M., Wu, Y., Wang, X., Wang, P., Zhang, L., Meng, X., Meng, F., Li, Y. (2023). Characterization of thioredoxin gene *TaTrxh9* associated with heading-time regulation in wheat. Plant Physiol. Biochem. 201:107903. [https://doi.org/10.1016/j.plaphy. .107903](https://doi.org/10.1016/j.plaphy.%20.107903)

Gupta, A., Hua, L., Zhang, Z., Yang, B., Li, W. (2023). CRISPR‐induced miRNA156‐recognition element mutations in *TaSPL13* improve multiple agronomic traits in wheat. Plant Biotechnol. J. 21:536. https://doi.org/10.1111/pbi.13969

Hahn, F., Sanjurjo Loures, L., Sparks, C. A., Kanyuka, K., Nekrasov, V. (2021). Efficient CRISPR/Cas-Mediated Targeted Mutagenesis in Spring and Winter Wheat Varieties. Plants. 10. https://doi.org/10.3390/plants10071481

He, F., Wang, C., Sun, H., Tian, S., Zhao, G., Liu, C., Wan, C., Guo, J., Huang, X., Zhan, G. (2023). Simultaneous editing of three homoeologues of *TaCIPK14* confers broad‐spectrum resistance to stripe rust in wheat. Plant Biotechnol. J. 21, 354-368. https://doi.org/10.1111/pbi.13956

Hyde, L., Osman, K., Winfield, M., Sanchez-Moran, E., Higgins, J. D., Henderson, I. R., Sparks, C., Franklin, F. C. H., Edwards, K. J. (2023). Identification, characterization, and rescue of CRISPR/Cas9 generated wheat SPO11-1 mutants. Plant Biotechnol. J. 21, 405-418. <https://doi.org/10.1111/pbi.13961>

Ibrahim, S., Saleem, B., Rehman, N., Zafar, S. A., Naeem, M. K., Khan, M. R. (2022). CRISPR/Cas9 mediated disruption of *Inositol Pentakisphosphate 2-Kinase 1* (*TaIPK1*) reduces phytic acid and improves iron and zinc accumulation in wheat grains. J. Adv. Res. 37:33. <https://doi.org/10.1016/j.jare.2021.07.006>

Jouanin, A., Borm, T., Boyd, L. A., Cockram, J., Leigh, F., Santos, B. A. C. M., Visser, R. G. F., Smulders, M. J. M. (2019). Development of the GlutEnSeq capture system for sequencing gluten gene families in hexaploid bread wheat with deletions or mutations induced by γ-irradiation or CRISPR/Cas9. J. Cereal Sci. 88:57. https://doi.org/10.1016/j.jcs.2019.04.008

Kan, J., Cai, Y., Cheng, C., Chen, S., Jiang, C., He, Z., Yang, P. (2023). CRISPR/Cas9-guided knockout of *eIF4E* improves Wheat yellow mosaic virus resistance without yield penalty. Plant Biotechnol. J. 21, 893-895. https://doi.org/10.1111/pbi.14002

Kong, X., Wang, F., Wang, Z., Gao, X., Geng, S., Deng, Z., Zhang, S., Fu, M., Cui, D., Liu, S. (2023). Grain yield improvement by genome editing of *TaARF12* that decoupled peduncle and rachis development trajectories via differential regulation of gibberellin signalling in wheat. Plant Biotechnol. J. 21, 1990-2001. https://doi.org/10.1111/pbi.14107

Li, H., Liu, H., Hao, C., Li, T., Liu, Y., Wang, X., Yang, Y., Zheng, J., Zhang, X. (2023a). The auxin response factor *TaARF15-A1* negatively regulates senescence in common wheat (*Triticum aestivum* L.). Plant Physiol. 191, 1254-1271. https://doi.org/10.1093/plphys/kiac497

Li, S., Lin, D., Zhang, Y., Deng, M., Chen, Y., Lv, B., Li, B., Lei, Y., Wang, Y., Zhao, L., Liang, Y., Liu, J., Chen, K., Liu, Z., Xiao, J., Qiu, J. L., Gao, C. (2022b). Genome-edited powdery mildew resistance in wheat without growth penalties. Nature. 602, 455-460. https://doi.org/10.1038/s41586-022-04395-9

Liu, S., Zhang, F., Su, J., Fang, A., Tian, B., Yu, Y., Bi, C., Ma, D., Xiao, S., Yang, Y. (2024c). CRISPR‐targeted mutagenesis of *mitogen‐activated protein kinase phosphatase 1* improves both immunity and yield in wheat. Plant Biotechnol. J. 22, 1929–1941. <https://doi.org/10.1111/pbi.14312>

Mohr, T., Horstman, J., Gu, Y. Q., Elarabi, N. I., Abdallah, N. A., Thilmony, R. (2022). CRISPR-Cas9 Gene Editing of the *Sal1* Gene Family in Wheat. Plants. 11. http://doi.org/10.3390/plants11172259

Rahim, A. A., Uzair, M., Rehman, N., Fiaz, S., Attia, K. A., Abushady, A. M., Yang, S. H., Khan, M. R. (2024). CRISPR/Cas9 mediated *TaRPK1* root architecture gene mutagenesis confers enhanced wheat yield. J. King Saud. Univ. Sci. 36:103063. <https://doi.org/10.1016/j.jksus.2023.103063>

Sun, W., Zhang, H., Yang, S., Liu, L., Xie, P., Li, J., Zhu, Y., Ouyang, Y., Xie, Q., Zhang, H., Yu, F. (2023b). Genetic modification of *Gγ subunit AT1* enhances salt-alkali tolerance in main graminaceous crops. Natl. Sci. Rev. 10(6). https://doi.org/10.1093/nsr/nwad075

Wang, W., Pan, Q., Tian, B., He, F., Chen, Y., Bai, G., Akhunova, A., Trick, H. N., Akhunov, E. (2019). Gene editing of the wheat homologs of *TONNEAU 1*‐recruiting motif encoding gene affects grain shape and weight in wheat. Plant J. 100, 251-264. https://doi.org/10.1111/tpj.14440

Wold-McGimsey, F., Krosch, C., Alarcón-Reverte, R., Ravet, K., Katz, A., Stromberger, J., Mason, R.E., Pearce, S. (2023). Multi-target genome editing reduces Polyphenol oxidase activity in wheat (*Triticum aestivum* L.) grains. Front. Plant Sci. 14:1247680. https://doi.org/10.3389/fpls.2023.1247680

Zhang, J., Zhang, H., Li, S., Li, J., Yan, L., Xia, L. (2021b). Increasing yield potential through manipulating of an *ARE1* ortholog related to nitrogen use efficiency in wheat by CRISPR/Cas9. J. Integr. Plant Biol. 63, 1649-1663. https://doi.org/10.1111/jipb.13151

Zhang, S., Zhang, R., Gao, J., Song, G., Li, J., Li, W., Qi, Y., Li, Y., Li, G. (2021c). CRISPR/Cas9‐mediated genome editing for wheat grain quality improvement. Plant Biotechnol. J. 19:1684. https://doi.org/10.1111/pbi.13647

Zhang, Z., Hua, L., Gupta, A., Tricoli, D., Edwards, K. J., Yang, B., Li, W. (2019b). Development of an *Agrobacterium*‐delivered CRISPR/Cas9 system for wheat genome editing. Plant Biotechnol. J. 17, 1623-1635. https://doi.org/10.1111/pbi.13088

Zhu, Y., Lin, Y., Fan, Y., Wang, Y., Li, P., Xiong, J., He, Y., Cheng, S., Ye, X., Wang, F., Goodrich, J., Zhu, J. K., Wang, K., Zhang, C. J. (2023). CRISPR/Cas9-mediated restoration of *Tamyb10* to create pre-harvest sprouting-resistant red wheat. Plant Biotechnol. J. 21, 665-667. https://doi.org/10.1111/pbi.13981

## References in Table 2: CRISPR/Cas9-mediated gene editing in Rice.

Achary, V. M. M., and Reddy, M. K. (2021). CRISPR-Cas9 mediated mutation in GRAIN WIDTH and WEIGHT2 (GW2) locus improves aleurone layer and grain nutritional quality in rice. Sci. Rep. 11:21941. <http://dx.doi.org/10.1038/s41598-021-00828-z>

Alam, M. S., Kong, J., Tao, R., Ahmed, T., Alamin, M., Alotaibi, S. S., Abdelsalam, N. R., Xu, J. H. (2022). CRISPR/Cas9 Mediated Knockout of the *OsbHLH024* Transcription Factor Improves Salt Stress Resistance in Rice (*Oryza sativa* L.). Plants. 11:1184. https://doi.org/10.3390/plants11091184

Alfatih, A. Wu, J., Jan, S. U., Zhang, Z. S., Xia, J. Q., Xiang, C. B. (2020). Loss of rice *PARAQUAT* *TOLERANCE 3* confers enhanced resistance to abiotic stresses and increases grain yield in field. Plant Cell Environ. 43, 2743-2754. <https://doi.org/10.1111/pce.13856>

Anjala, K., and Augustine, R. (2022), Designing of guide RNA constructs for CRISPR/Cas9-mediated editing of rice transcription factor osmads26 for enhancing drought tolerance. J. Appl. Biol. Biotechnol. 11:176. https://doi.org/10.7324/jabb.2023.110124

Biswas, S., Ibarra, O., Shaphek, M., Molina-Risco, M., Faion-Molina, M., Bellinatti-Della Gracia, M., Thomson, M. J., Septiningsih, E. M. (2023). Increasing the level of resistant starch in ‘Presidio’ rice through multiplex CRISPR–Cas9 gene editing of starch branching enzyme genes. TPG. 16:e20225. https://doi.org/10.1002/tpg2.20225

Caddell, D., Langenfeld, N. J., Eckels, M. J. H., Zhen, S., Klaras, R., Mishra, L., Bugbee, B., Coleman-Derr, D. (2023). Photosynthesis in rice is increased by CRISPR/Cas9-mediated transformation of two truncated light-harvesting antenna. Front. Plant Sci. 14. <https://doi.org/10.3389/fpls.2023.1050483>

Chen, H., Ye, R., Liang, Y., Zhang, S., Liu, X., Sun, C., Li, F., Yi, J. (2023). Generation of low-cadmium rice germplasms via knockout of *OsLCD* using CRISPR/Cas9. J. Environ. Sci. 126, 138-152. https://doi.org/10.1016/j.jes.2022.05.047

Chen, W., Chen, L., Zhang, X., Yang, N., Guo, J., Wang, M., Ji, S., Zhao, X., Yin, P., Cai, L., Xu, J., Zhang, L., Han, Y., Xiao, Y., Xu, G., Wang, Y., Wang, S., Wu, S., Yang, F., et al. (2022b). Convergent selection of a WD40 protein that enhances grain yield in maize and rice. Science, 375(6587). https://doi.org/10.1126/science.abg7985

Chen, X., Guo, Q., Yang, X., Yuan, M., Song, J., Fu, H., Zhang, H., Xu, P., Liao, Y., Ali, A., Du, K. and Wu, X. (2024). Triple gene mutations boost amylose and resistant starch content in rice: insights from *sbe2b/sbe1/OE-Wxa* mutants. Front. Plant Sci. 15:1452520. https://doi.org/10.3389/fpls.2024.1452520

Chen, Z., Du, H., Tao, Y., Xu, Y., Wang, F., Li, B., Zhu, Q. H., Niu, H., Yang, J. (2022c). Efficient breeding of low glutelin content rice germplasm by simultaneous editing multiple glutelin genes via CRISPR/Cas9. Plant Sci. 324:111449. <https://doi.org/10.1016/j.plantsci.2022.111449>

Ding, Y., Zhang, F., Sun, F., Liu, J., Zhu, Z., He, X., Bai, G., Ni, Z., Sun, Q., and Su, Z. (2023). Loss of *OsHRC* function confers blast resistance without yield penalty in rice. Plant Biotechnol. J. 21(8), 1516–1518. https://doi.org/10.1111/pbi.14061

Fu, K., Song, W., Chen, C., Mou, C., Huang, Y., Zhang, F., Hao, Q., Wang, P., Ma, T., Chen, Y., Zhu, Z., Zhang, M., Tong, Q., Liu, X., Jiang, L., Wan, J. (2022). Improving pre-harvest sprouting resistance in rice by editing *OsABA8ox* using CRISPR/Cas9*.* Plant Cell Rep. 41, 2107-2110. <https://doi.org/10.1007/s00299-022-02917-3>

Gao, X., Li, J., Yin, J., Zhao, Y., Wu, Z., Ma, L., Zhang, B., Zhang, H., and Huang, J. (2024). The protein phosphatase *qGL3/OsPPKL1* self-regulates its degradation to orchestrate brassinosteroid signaling in rice. Plant Commun. 5(6). https://doi.org/10.1016/j.xplc.2024.100849

Huang, Q., Lin, B., Cao, Y., Zhang, Y., Song, H., Huang, C., Sun, T., Long, C., Liao, J., Zhuo, K. (2023). CRISPR/Cas9-mediated mutagenesis of the susceptibility gene *OsHPP04* in rice confers enhanced resistance to rice root-knot nematode. Front. Plant Sci. 14. <https://doi.org/10.3389/fpls.2023.1134653>

Hui, S., Li, H., Mawia, A. M., Zhou, L., Cai, J., Ahmad, S., Lai, C., Wang, J., Jiao, G., Xie, L. (2022). Production of aromatic three‐line hybrid rice using novel alleles of *BADH2*. Plant Biotechnol. J. 20:59. <https://doi.org/10.1111/pbi.13695>

Iwamoto, M. (2022). In-frame editing of transcription factor gene *RDD1* to suppress miR166 recognition influences nutrient uptake, photosynthesis, and grain quality in rice. Sci. Rep. 12:10795. https://doi.org/10.1038/s41598-022-14768-9

Jameel, M. R., Ansari, Z., Al-Huqail, A. A., Naaz, S., Qureshi, M. I. (2022). CRISPR/Cas9-Mediated Genome Editing of Soluble Starch Synthesis Enzyme in Rice for Low Glycemic Index. Agronomy. 2. <https://doi.org/10.3390/agronomy12092206>

Jiang, M., Zhang, H., Song, Y., Chen, J., Bai, J., Tang, J., Wang, Q., Fotopoulos, V., Qian-Hao Zhu, Yang, R., and Li, R. (2024b). Transcription factor *OsbZIP10* modulates rice grain quality by regulating *OsGIF1*. Plant J. 119(5), 2181–2198. <https://doi.org/10.1111/tpj.16911>

Jin, S. K., Xu, L. N., Leng, Y. J., Zhang, M. Q., Yang, Q. Q., Wang, S. L., Jia, S. W., Song, T., Wang, R. A., Tao, T., Liu, Q. Q., Cai, X. L., Gao, J. P. (2023), The *OsNAC24-OsNAP* protein complex activates *OsGBSSI* and *OsSBEI* expression to fine-tune starch biosynthesis in rice endosperm. Plant Biotechnol. J. 21: 2224-2240. <https://doi.org/10.1111/pbi.14124>

Kim, J. Y., Lee, Y. J., Lee, H. J., Go, J. Y., Lee, H. M., Park, J. S., Cho, Y. G., Jung, Y. J., Kang, K. K. (2024). Knockout of *OsGAPDHC7* Gene Encoding Cytosolic Glyceraldehyde- 3-Phosphate Dehydrogenase Affects Energy Metabolism in Rice Seeds. Int. J. Mol. Sci. 25, 12470. https://doi.org/10.3390/ijms252212470

Kuai, P., Lin, N., Ye, M. et al. (2024). Identification and knockout of a herbivore susceptibility gene enhances planthopper resistance and increases rice yield. Nat. Food 5, 846–859. https://doi.org/10.1038/s43016-024-01044-4

Li, B., Du, X., Fei, Y., Wang, F., Xu, Y., Li, X., Li, W., Chen, Z., Fan, F., Wang, J., Tao, Y., Jiang, Y., Zhu, Q. H., Yang, J. (2021a). Efficient Breeding of Early-Maturing Rice Cultivar by Editing *PHYC* via CRISPR/Cas9. Rice. 14:86. https://doi.org/10.1186/s12284-021-00527-3

Li, H., Zhang, Y., Wu, C., Bi, J., Chen, Y., Jiang, C., Cui, M., Chen, Y., Hou, X., Yuan, M., Xiong, L., Yang, Y., Xie, K. (2022a). Fine-tuning *OsCPK18/OsCPK4* activity via genome editing of phosphorylation motif improves rice yield and immunity. Plant Biotechnol. J. 20, 2258-2271. <https://doi.org/10.1111/pbi.13905>

Li, X., Yu, Y., Yao, W., Yin, Z., Wang, Y., Huang, Z., Zhou, J. Q., Liu, J., Lu, X., Wang, F. (2023b). CRISPR/Cas9‐mediated simultaneous mutation of three *salicylic acid 5‐hydroxylase* (*OsS5H*) genes confers broad‐spectrum disease resistance in rice. Plant Biotechnol. J. 21:1873. https://doi.org/10.1111/pbi.14099

Li, Y., Wu, S., Huang, Y., Ma, X., Tan, L., Liu, F., Lv, Q., Zhu, Z., Hu, M., Fu, Y., Zhang, K., Gu, P., Xie, D., Sun, H., and Sun, C. (2023c). *OsMADS17* simultaneously increases grain number and grain weight in rice. Nat. Commun. 14(1), 3098. https://doi.org/10.1038/s41467-023-38726-9

Li, Z., Rao, M. J., Li, J., Wang, Y., Chen, P., Yu, H., Ma, C., Wang, L. (2022c). CRISPR/Cas9 Mutant Rice *Ospmei12* Involved in Growth, Cell Wall Development, and Response to Phytohormone and Heavy Metal Stress. Int. J. Mol. Sci. 23. https://doi.org/10.3390/ijms232416082

Liao, S., Qin, X., Luo, L., Han, Y., Wang, X., Usman, B., Nawaz, G., Zhao, N., Liu, Y., Li, R. (2019). CRISPR/Cas9-induced mutagenesis of *semi-rolled leaf1, 2* confers curled leaf phenotype and drought tolerance by influencing protein expression patterns and ROS scavenging in rice (*Oryza sativa* L.). Agronomy. 9:728. https://doi.org/10.3390/agronomy9110728

Liu, K., Sakuraba, Y., Ohtsuki, N., Yang, M., Ueda, Y., Yanagisawa, S. (2023a). CRISPR/Cas9-mediated elimination of *OsHHO3*, a transcriptional repressor of three *AMMONIUM TRANSPORTER1* genes, improves nitrogen use efficiency in rice. Plant Biotechnol. J. 21, 2169-2172. https://doi.org/10.1038/s41477-021-00858-5

Liu, X., Ding, Q., Wang, W., Pan, Y., Tan, C., Qiu, Y., Chen, Y., Li, H., Li, Y., Ye, N., Xu, N., Wu, X., Ye, R., Liu, J., Ma, C. (2022b). Targeted Deletion of the First Intron of the *Wxb* Allele via CRISPR/Cas9 Significantly Increases Grain Amylose Content in Rice. Rice. 15:1. https://doi.org/10.1186/s12284-021-00548-y

Lu, Q., Luo, X., Yang, X., Zhou, T., Zhang, Y., Lan, Y., Zhang, D., Zheng, L., Li, Y., Li, L., Zhang, S., Liu, Y. (2023). CRISPR/Cas9-mediated gene editing of vacuolar *ATPase subunit d* mediates phytohormone biosynthesis and virus resistance in rice. Front. Plant Sci. 14. https://doi.org/10.3389/fpls.2023.1122978

Ly, L. K., Ho, T. M., Bui, T. P., Nguyen, L. T., Phan, Q., Le, N. T., Khuat, L. T. M., Le, L. H., Chu, H. H., Pham, N. B., Do, P. T. (2024). CRISPR/Cas9 targeted mutations of *OsDSG1* gene enhanced salt tolerance in rice. Funct. Integr. Genom. 24:70. <https://doi.org/10.1007/s10142-024-01347-6>

Peng, B., Liu, Y., Sun, X., Zhao, Q., Qiu, J., Tian, X., Peng, J., Zhang, Z., Wang, Y., Huang, Y., Pang, R., Zhou, W., Qi, Y., Sun, Y., Wang, Q., He, Y. (2024) The *OsGAPC3* mutation significantly affects grain quality traits and improves the nutritional quality of rice. Front. Plant Sci. 15:1470316. https://doi.org/10.3389/fpls.2024.1470316

Qin, Q., Wang, Y., Huang, L., Du, F., Zhao, X., Li, Z., Wang, W., Fu, B. (2020). A *U-box E3 ubiquitin ligase* *OsPUB67* is positively involved in drought tolerance in rice. Plant Mol. Biol. 102, 89-107. https://doi.org/10.1007/s11103-019-00933-8

Qing, D., Chen, W., Huang, S., Li, J., Pan, Y., Zhou, W., Liang, Q., Yuan, J., Gan, D., Chen, L., Chen, L., Huang, J., Zhou, Y., Dai, G., Deng, G. (2023). Editing of rice (*Oryza sativa* L.) *OsMKK3* gene using CRISPR/Cas9 decreases grain length by modulating the expression of photosystem components. PROTEOMICS. 23:2200538. https://doi.org/10.1002/pmic.202200538

Shah, P. R., Varanavasiappan, S., Kokiladevi, E., Ramanathan, A., Kumar, K. K. (2019). Genome editing of rice *PFT1* gene to study its role in rice sheath blight disease resistance. Int. J. Curr. Microbiol. Appl. Sci. 8, 2356-2364. https://doi.org/10.20546/ijcmas.2019.806.281

Sheng, X., Ai, Z., Tan, Y., Hu, Y., Guo, X., Liu, X., Sun, Z., Yu, D., Chen, J., Tang, N., Duan, M., Yuan, D. (2023). Novel Salinity-Tolerant Third-Generation Hybrid Rice Developed via CRISPR/Cas9-Mediated Gene Editing. Int. J. Mol. Sci. 24. <https://doi.org/10.3390/ijms24098025>

Shim, K. C., Adeva, C., Kang, J. W., Luong, N. H., Lee, H. S., Cho, J. H., Kim, H., Tai, T. H., Ahn, S. N. (2022). Interaction of *starch branching enzyme 3* and *granule-bound starch synthase 1* alleles increases amylose content and alters physico-chemical properties in japonica rice (*Oryza sativa* L.). Front. Plant Sci. 13:968795. https://doi.org10.3389/fpls.2022.968795

Shim, Y., Seong, G., Choi, Y., Lim, C., Baek, S. A., Park, Y. J., Kim, J. K., An, G., Kang, K., Paek, N. C. (2023). Suppression of cuticular wax biosynthesis mediated by rice *LOV KELCH REPEAT* *PROTEIN 2* supports a negative role in drought stress tolerance. Plant Cell Environ. 46, 1504-1520. https://doi.org/10.1111/pce.14549

Song, X., Chen, Z., Du, X., Li, B., Fei, Y., Tao, Y., Wang, F., Xu, Y., Li, W., Wang, J., Liang, G., Zhou, Y., Tan, X., Li, Y., Yang, J. (2023). Generation of new rice germplasms with low amylose content by CRISPR/CAS9-targeted mutagenesis of the *FLOURY ENDOSPERM 2* gene. Front. Plant Sci. 14. <https://doi.org/10.3389/fpls.2023.1138523>

Sony, S. K., Kaul, T., Motelb, K. F. A., Thangaraj, A., Bharti, J., Kaul, R., Verma, R., Nehra, M. (2023). CRISPR/Cas9‐mediated homology donor repair base editing confers glyphosate resistance to rice (*Oryza sativa L*.). Front. Plant Sci. 14:1122926. https://doi.org/10.3389/fpls.2023.1122926

Sun, W., Zhang, H., Yang, S., Liu, L., Xie, P., Li, J., Zhu, Y., Ouyang, Y., Xie, Q., Zhang, H., Yu, F. (2023). Genetic modification of *Gγ subunit AT1* enhances salt-alkali tolerance in main graminaceous crops. Natl. Sci. Rev. 10(6). https://doi.org/10.1093/nsr/nwad075

Távora, F. T. P. K., Meunier, A. C., Vernet, A., Portefaix, M., Milazzo, J., Adreit, H., Tharreau, D., Franco, O. L., Mehta, A. (2022). CRISPR/Cas9-Targeted knockout of rice susceptibility genes *OsDjA2* and *OsERF104* reveals alternative sources of resistance to *Pyricularia oryzae*. Rice Sci. 29, 535-544. <https://doi.org/10.1016/j.rsci.2022.04.001>

Tu, B., Zhang, T., Liu, P., Yang, W., Zheng, L., Dai, Y., Wang, H., Lin, S., Zhang, Z., Zheng, X., Yuan, M., Chen, Y., Zhu, X., Yuan, H., Li, T., Xiong, J., Zhong, Z., Chen, W., Ma, B., et al. (2024). The *LCG1-OsBP5/OsEBP89-Wx* module regulates the grain chalkiness and taste quality in rice. Plant Biotechnol. J. 23(1), 36–50. https://doi.org/10.1111/pbi.14475

Usman, B., Nawaz, G., Zhao, N., Liao, S., Qin, B., Liu, F., Liu, Y., Li, R. (2021). Programmed Editing of Rice (*Oryza sativa L*.) *OsSPL16* Gene Using CRISPR/Cas9 Improves Grain Yield by Modulating the Expression of Pyruvate Enzymes and Cell Cycle Proteins. Int. J. Mol. Sci. 22. https://doi.org/10.3390/ijms22010249.

Usman, B., Nawaz, G., Zhao, N., Liu, Y., Li, R. (2020). Generation of high yielding and fragrant rice (*Oryza sativa* L.) lines by CRISPR/Cas9 targeted mutagenesis of three homoeologs of cytochrome *P450* gene family and *OsBADH2* and transcriptome and proteome profiling of revealed changes triggered by mutations. Plants. 9:788. https://doi.org/10.3390/plants9060788

Wang, B., Zhong, Z., Wang, X., Han, X., Yu, D., Wang, C., Song, W., Zheng, X., Chen, C., Zhang, Y. (2020a). Knockout of the *OsNAC006* transcription factor causes drought and heat sensitivity in rice. Int. J. Mol. Sci. 21:2288. <https://doi.org/10.3390/ijms21072288>

Wang, C., Wang, G., Gao, Y., Lu, G., Habben, J. E., Mao, G., Chen, G., Wang, J., Yang, F., Zhao, X. (2020b) A cytokinin-activation enzyme-like gene improves grain yield under various field conditions in rice. Plant Mol. Biol. 102, 373-388. <https://doi.org/10.1007/s11103-019-00952-5>

Wang, J. D., Wang, J., Huang, L. C., Kan, L. J., Wang, C. X., Xiong, M., Zhou, P., Zhou, L. H., Chen, C., Zhao, D. S., Fan, X. L., Zhang, C. Q., Zhou, Y., Zhang, L., Liu, Q. Q., Li, Q. F. (2024c). ABA-mediated regulation of rice grain quality and seed dormancy via the *NF-YB1-SLRL2-bHLH144* Module. Nat. Commun. 15(1), 4493. https://doi.org/10.1038/s41467-024-48760-w

Wang, W., Wang, W., Pan, Y., Tan, C., Li, H., Chen, Y., Liu, X., Wei, J., Xu, N., Han, Y., Gu, H., Ye, R., Ding, Q., Ma, C. (2022a). A new gain-of-function *OsGS2/GRF4* allele generated by CRISPR/Cas9 genome editing increases rice grain size and yield. Crop J. 10, 1207-1212. <https://doi.org/10.1016/j.cj.2022.01.004>

Wang, X., Zhang, Z., Peng, W., Huang, J., Yan, X., Yao, W., Ouyang, J., Li, S. (2023). *Inositolphosphorylceramide synthases*, *OsIPCSs*, regulate plant height in rice. Plant Sci. 335. https://doi.org/10.1016/j.plantsci.2023.111798

Wu, Q., Liu, Y., Huang, J. (2022). CRISPR-Cas9 Mediated Mutation in *OsPUB43* Improves Grain Length and Weight in Rice by Promoting Cell Proliferation in Spikelet Hull. Int. J. Mol. Sci. 23. https://doi.org/10.3390/ijms23042347

Wu, S., Kyaw, H., Tong, Z., Yang, Y., Wang, Z., Zhang, L., Deng, L., Zhang, Z., Xiao, B., Quick, W.P., Lu, T., Xiao, G., Qin, G., Cui, X.a. (2024). A simple and efficient CRISPR/Cas9 system permits ultra-multiplex genome editing in plants. Crop J. 12, 569–582. https://doi.org/10.1016/j.cj.2024.01.010

Wu, Y., Xiao, N., Cai, Y., Yang, Q., Yu, L., Chen, Z., Shi, W., Liu, J., Pan, C., Li, Y. (2023). CRISPR-Cas9-mediated editing of the *OsHPPD* 3′ UTR confers enhanced resistance to HPPD-inhibiting herbicides in rice. Plant Commun. 4. <https://doi.org/10.1016/j.xplc.2023.100605>

Xie, Z., Sun, Y., Zhan, C., Qu, C., Jin, N., Gu, X., Huang, J. (2024c). The *E3 ligase* *OsPUB33* controls rice grain size and weight by regulating the *OsNAC120-BG1* module. Plant Cell. 37(1). https://doi.org/10.1093/plcell/koae297

Xiong, D., Wang, R., Wang, Y., Li, Y., Sun, G., Yao, S. (2023). *SLG2* specifically regulates grain width through *WOX11*-mediated cell expansion control in rice. Plant Biotechnol. J. 21(9), 1904–1918. <https://doi.org/10.1111/pbi.14102>

Xu, H., Yang, X., Zhang, Y., Wang, H., Wu, S., Zhang, Z., Ahammed, G. J., Zhao, C., Liu, H. (2022). CRISPR/Cas9-mediated mutation in auxin efflux carrier *OsPIN9* confers chilling tolerance by modulating reactive oxygen species homeostasis in rice. Front. Plant Sci. 13. https://doi.org/10.3389/fpls.2022.967031

Yang, C. h., Zhang, Y., Huang, C. f. (2019). Reduction in cadmium accumulation in japonica rice grains by CRISPR/Cas9-mediated editing of *OsNRAMP5*. J. Integr. Agric. 18, 688-697. <https://doi.org/10.1016/S2095-3119(18)61904-5>

Yang, Y., Zhang, T., Shi, Y., Lu, Y., Li, Q., Fan, X., Huang, L., Chen, L., Song, X., Liu, Q., Zhang, C., Liu, Q. (2024). Introgression of *lac6/tl1/du13* improves the palatability of japonica rice. Crop J. 12(4), 1259–1265. https://doi.org/10.1016/j.cj.2024.06.006

Yang, Y., Zhang, Y., Sun, Z., Shen, Z., Li, Y., Guo, Y., Feng, Y., Sun, S., Guo, M., Hu, Z., Yan, C. (2023). Knocking Out *OsAAP11* to Improve Rice Grain Quality Using CRISPR/Cas9 System. Int. J. Mol. Sci. 24. https://doi.org/10.3390/ijms241814360

Yue, E., Cao, H., Liu, B. (2020). *OsmiR535*, a potential genetic editing target for drought and salinity stress tolerance in *Oryza sativa*. Plants. 9:1337. <https://doi.org/10.3390/plants9101337>

Yuyu, C., Aike, Z., Pao, X., Xiaoxia, W., Yongrun, C., Beifang, W., Yue, Z., Liaqat, S., Shihua, C., Liyong, C., Yingxin, Z. (2020). Effects of *GS3* and *GL3.1* for Grain Size Editing by CRISPR/*Cas9* in Rice. Rice Sci. 27:405. https://doi.org/10.1016/j.rsci.2019.12.010

Zafar, K., Khan, M. Z., Amin, I., Mukhtar, Z., Zafar, M., Mansoor, S. (2023). Employing template-directed CRISPR-based editing of the *OsALS* gene to create herbicide tolerance in Basmati rice. AoBP. 15(2), plac059. https://doi.org/10.1093/aobpla/plac059

Zeng, Y., Wen, J., Zhao, W., Wang, Q., Huang, W. (2020). Rational Improvement of Rice Yield and Cold Tolerance by Editing the Three Genes *OsPIN5b*, *GS3*, and *OsMYB30* With the CRISPR–Cas9 System. Front. Plant Sci. 10. https://doi.org/10.3389/fpls.2019.01663

Zhang, C., Yun, P., Xia, J., Zhou, K., Wang, L., Zhang, J., Zhao, B., Yin, D., Fu, Z., Wang, Y., Ma, T., Li, Z., Wu, D. (2023a). CRISPR/Cas9-mediated editing of *Wx* and *BADH2* genes created glutinous and aromatic two-line hybrid rice. Mol. Breed. 43:24. <https://doi.org/10.1007/s11032-023-01368-2>

Zhang, J., Augustine, R. C., Suzuki, M., Feng, J., Char, S. N., Yang, B., McCarty, D. R., Vierstra, R. D. (2021a). The *SUMO ligase MMS21* profoundly influences maize development through its impact on genome activity and stability. PLOS Genet. 17:e1009830.

Zhang, W., Wang, R., Kong, D., Peng, F., Chen, M., Zeng, W., Giaume, F., He, S., Zhang, H., Wang, Z., Kyozuka, J., Zhu, J. K., Fornara, F., Miki, D. (2023c). Precise and heritable gene targeting in rice using a sequential transformation strategy. Cell Rep. Methods. 3:100389. https://doi.org/10.1016/j.crmeth.2022.100389

Zhang, Y., Lin, X. F., Li, L., Piao, R. H., Wu, S., Song, A., Gao, M., Jin, Y. M. (2024). CRISPR/Cas9-mediated knockout of *Bsr-d1* enhances the blast resistance of rice in Northeast China. Plant Cell Rep. 43:100. https://doi.org/10.1007/s00299-024-03192-0

Zheng, S., Ye, C., Lu, J., Liufu, J., Lin, L., Dong, Z., Li, J., Zhuang, C. (2021b). Improving the Rice Photosynthetic Efficiency and Yield by Editing *OsHXK1* via CRISPR/Cas9 System. Int. J. Mol. Sci. 22. https://doi.org/10.3390/ijms22179554

Zhou, J., Zhang, R., Jia, X., Tang, X., Guo, Y., Yang, H., Zheng, X., Qian, Q., Qi, Y., Zhang, Y. (2024a). CRISPR-Cas9 mediated *OsMIR168a* knockout reveals its pleiotropy in rice. Plant Biotechnol. J. 20, 310-322. https://doi.org/10.1111/pbi.13713

Zhou, S., Cai, L., Wu, H., Wang, B., Gu, B., Cui, S., Huang, X., Xu, Z., Hao, B., Hou, H., Hu, Y., Li, C., Tian, Y., Liu, X., Chen, L., Liu, S., Jiang, L., Wan, J. (2024b). Fine-tuning rice heading date through multiplex editing of the regulatory regions of key genes by CRISPR-Cas9. Plant Biotechnol. J. 22, 751-758. https://doi.org/10.1111/pbi.14221

## References in Table 3: CRISPR/Cas9-mediated gene editing in Maize.

An, X., Zhang, S., Jiang, Y., Liu, X., Fang, C., Wang, J., Zhao, L., Hou, Q., Zhang, J., Wan, X. (2024). CRISPR/Cas9-based genome editing of 14 lipid metabolic genes reveals a sporopollenin metabolon *ZmPKSB-ZmTKPR1-1/-2* required for pollen exine formation in maize. Plant Biotechnol. J. 22, 216-232. https://doi.org/10.1111/pbi.14181

Chen, Z., Li, W., Gaines, C., Buck, A., Galli, M., Gallavotti, A. (2021). Structural variation at the maize *WUSCHEL1* locus alters stem cell organization in inflorescences. Nat. Commun. 12:2378. <https://doi.org/10.1038/s41467-021-22699-8>

Gao, L., Yang, G., Li, Y., Sun, Y., Xu, R., Chen, Y., Wang, Z., Xing, J., Zhang, Y. (2021). A kelch-repeat superfamily gene, *ZmNL4*, controls leaf width in maize (*Zea mays* L.). Plant J. 107:817. <https://doi.org/10.1111/tpj.15348>

Guan, H., Chen, X., Wang, K., Liu, X., Zhang, D., Li, Y., Song, Y., Shi, Y., Wang, T., Li, C. (2022). Genetic variation in *ZmPAT7* contributes to tassel branch number in maize. Int. J. Mol. Sci. 23:2586. <https://doi.org/10.3390/ijms23052586>

Jia, H., Li, M., Li, W., Liu, L., Jian, Y., Yang, Z., Shen, X., Ning, Q., Du, Y., Zhao, R. (2020). A serine/threonine protein kinase encoding gene *KERNEL NUMBER PER ROW6* regulates maize grain yield. Nat. Commun. 11:988. <https://doi.org/10.1038/s41467-020-14746-7>

Jiang, L., Guo, T., Song, X., Jiang, H., Lu, M., Luo, J., Rossi, V., He, Y. (2024a). *MSH7* confers quantitative variation in pollen fertility and boosts grain yield in maize. Plant Biotechnol. J. 22:1372. https://doi.org/10.1111/pbi.14272

Jing, T., Wu, Y., Yu, Y., Li, J., Mu, X., Xu, L., Wang, X., Qi, G., Tang, J., Wang, D., Yang, S., Hua, J., Gou, M. (2024b). Copine proteins are required for brassinosteroid signaling in maize and *Arabidopsis*. Nat. Commun. 15:2028. <https://doi.org/10.1038/s41467-024-46289-6>

Kaul, T., Thangaraj, A., Jain, R., Bharti, J., Kaul, R., Verma, R., Sony, S. K., Abdel Motelb, K. F., Yadav, P., Agrawal, P. K. (2024). CRISPR/Cas9-mediated homology donor repair base editing system to confer herbicide resistance in maize (*Zea mays* L.). Plant Physiol. Biochem. 207:108374. https://doi.org/10.1016/j.plaphy.2024.108374

Li, W., Li, Y., Shi, H., Wang, H., Ji, K., Zhang, L., Wang, Y., Dong, Y., Li, Y. (2024b). *ZmMPK6*, a *mitogen-activated protein kinase*, regulates maize kernel weight. J. Exp. Bot. erae104. <https://doi.org/10.1093/jxb/erae104>

Liu, B., Wang, N., Yang, R., Wang, X., Luo, P., Chen, Y., Wang, F., Li, M., Weng, J., Zhang, D., Yong, H., Han, J., Zhou, Z., Zhang, X., Hao, Z., Li, X. (2024a). *ZmADF5*, a Maize Actin-Depolymerizing Factor Conferring Enhanced Drought Tolerance in Maize. Plants. 13: 619. <https://doi.org/10.3390/plants13050619>

Liu, C., Kong, M., Yang, F., Zhu, J., Qi, X., Weng, J., Di, D., Xie, C. (2022a). Targeted generation of Null Mutants in *ZmGDIα* confers resistance against maize rough dwarf disease without agronomic penalty. Plant Biotechnol. J. 20:803. <https://doi.org/10.1111/pbi.13793>

Liu, L., Gallagher, J., Arevalo, E. D., Chen, R., Skopelitis, T., Wu, Q., Bartlett, M., Jackson, D. (2021). Enhancing grain-yield-related traits by CRISPR–Cas9 promoter editing of maize *CLE* genes. Nat. Plants. 7:287. <https://doi.org/10.1038/s41477-021-00858-5>

Liu, X., Zhang, S., Jiang, Y., Yan, T., Fang, C., Hou, Q., Wu, S., Xie, K., An, X., Wan, X. (2022c). Use of CRISPR/Cas9-Based Gene Editing to Simultaneously Mutate Multiple Homologous Genes Required for Pollen Development and Male Fertility in Maize. Cells. 11. <https://doi.org/10.3390/cells11030439>

Liu, Y., Chen, Z., Zhang, C., Guo, J., Liu, Q., Yin, Y., Hu, Y., Xia, H., Li, B., Sun, X., Li, Y., Liu, X. (2023b). Gene editing of *ZmGA20ox3* improves plant architecture and drought tolerance in maize. Plant Cell Rep, 43:18. <https://doi.org/10.1007/s00299-023-03090-x>

Lv, G., Li, Y., Wu, Z., Zhang, Y., Li, X., Wang, T., Ren, W., Liu, L., Chen, J., Zhang, Y. (2024). *Maize actin depolymerizing factor 1* (*ZmADF1*) negatively regulates pollen development. BBRC 703:149637. <https://doi.org/10.1016/j.bbrc.2024.149637>

Ma, L., Sun, Y., Ruan, X., Huang, P. C., Wang, S., Li, S., Zhou, Y., Wang, F., Cao, Y., Wang, Q. (2021). Genome-wide characterization of jasmonates signaling components reveals the essential role of *ZmCOI1a-ZmJAZ15* action module in regulating maize immunity to gibberella stalk rot. Int. J. Mol. Sci. 22:870. <https://doi.org/10.3390/ijms22020870>

Ning, Q., Jian, Y., Du, Y., Li, Y., Shen, X., Jia, H., Zhao, R., Zhan, J., Yang, F., Jackson, D. (2021). An ethylene biosynthesis enzyme controls quantitative variation in maize ear length and kernel yield. Nat. Commun. 12:5832. https://doi.org/10.1038/s41467-021-26123-z

Pan, Z., Liu, M., Zhao, H., Tan, Z., Liang, K., Sun, Q., Gong, D., He, H., Zhou, W., Qiu, F. (2020b). *ZmSRL5* is involved in drought tolerance by maintaining cuticular wax structure in maize. J. Integr. Plant Biol. 62, 1895-1909. <https://doi.org/10.1111/jipb.12982>

Pang, Y., Cao, L., Ye, F., Ma, C., Liang, X., Song, Y., Lu, X. (2024). Identification of the Maize *PP2C* Gene Family and Functional Studies on the Role of *ZmPP2C15* in Drought Tolerance. Plants. 13. <https://doi.org/10.3390/plants13030340>

Qi, X., Guo, S., Wang, D., Zhong, Y., Chen, M., Chen, C., Cheng, D., Liu, Z., An, T., Li, J. (2022). *ZmCOI2a* and *ZmCOI2b* redundantly regulate anther dehiscence and gametophytic male fertility in maize. Plant J. 110:849. https://doi.org/10.1111/tpj.15708

Ren, R. C., Kong, L. G., Zheng, G. M., Zhao, Y. J., Jiang, X., Wu, J. W., Liu, C., Chu, J., Ding, X. H., Zhang, X. S., Wang, G. F., Zhao, X. Y. (2024). Maize requires *arogenate dehydratase 2* for resistance to *Ustilago maydis* and plant development. Plant Physiol. kiae115. <https://doi.org/10.1093/plphys/kiae115>

Wang, Y., Tang, Q., Kang, Y., Wang, X., Zhang, H., Li, X. (2022b). Analysis of the utilization and prospects of CRISPR-Cas technology in the annotation of gene function and creation new germplasm in maize based on patent data. Cells. 11:3471. <https://doi.org/10.3390/cells11213471>

Xie, J., Fei, X., Yan, Q., Jiang, T., Li, Z., Chen, H., Wang, B., Chao, Q., He, Y., Fan, Z., Wang, L., Wang, M., Shi, L., Zhou, T. (2024a). The C4 photosynthesis bifunctional enzymes, PDRPs, of maize are co-opted to cytoplasmic viral replication complexes to promote infection of a prevalent potyvirus sugarcane mosaic virus. Plant Biotechnol. J. 22:1812. https://doi.org/10.1111/pbi.14304

Xie, Y., Zhao, Y., Chen, L., Wang, Y., Xue, W., Kong, D., Li, C., Zhou, L., Li, H., Zhao, Y., Wang, B., Xu, M., Zhao, B., Bilska-Kos, A. and Wang, H. (2024b). *ZmELF3.1* integrates the RA2-TSH4 module to repress maize tassel branching. New Phytol. 241:490-503.

Xu, R., Li, Y., Sui, Z., Lan, T., Song, W., Zhang, M., Zhang, Y., Xing, J. (2021). A C-terminal encoded peptide, *ZmCEP1*, is essential for kernel development in maize. J. Exp. Bot. 72:5390. https://doi.org/10.1093/jxb/erab224

Yan, Z., Li, K., Li, Y., Wang, W., Leng, B., Yao, G., Zhang, F., Mu, C., Liu, X. (2023). The *ZmbHLH32-ZmIAA9-ZmARF1* module regulates salt tolerance in maize. Int. J. Biol. Macromol. 253:126978. <https://doi.org/10.1016/j.ijbiomac.2023.126978>

Zhang, Q., Tian, S., Chen, G., Tang, Q., Zhang, Y., Fleming, A. J., Zhu, X. G., Wang, P. (2023b). A regulatory circuit involving the NADH dehydrogenase-like complex balances C4 photosynthetic carbon flow and cellular redox in maize [Preprint]. Available at: https://www.biorxiv.org/content/10.1101/2023.02.23.529632v1. (Accessed October 15, 2024).

Zhong, T., Zhu, M., Zhang, Q., Zhang, Y., Deng, S., Guo, C., Xu, L., Liu, T., Li, Y., Bi, Y., Fan, X., Balint-Kurti, P., Xu, M. (2024b). The *ZmWAKL*–*ZmWIK*–*ZmBLK1*–*ZmRBOH4* module provides quantitative resistance to gray leaf spot in maize. Nat. Genet. 56, 315-326. https://doi.org/10.1038/s41588-023-01644-z
